# Analysis of 1508 Plasma Samples by Capillary-Flow Data-Independent Acquisition Profiles Proteomics of Weight Loss and Maintenance*[Fn FN2]

**DOI:** 10.1074/mcp.RA118.001288

**Published:** 2019-04-04

**Authors:** Roland Bruderer, Jan Muntel, Sebastian Müller, Oliver M. Bernhardt, Tejas Gandhi, Ornella Cominetti, Charlotte Macron, Jérôme Carayol, Oliver Rinner, Arne Astrup, Wim H.M. Saris, Jörg Hager, Armand Valsesia, Loïc Dayon, Lukas Reiter

**Affiliations:** From the ‡Biognosys, 8952 Zurich-Schlieren, Switzerland;; §Nestlé Institute of Health Sciences, 1015 Lausanne, Switzerland;; ¶Department of Nutrition, Exercise and Sports, Faculty of Science, University of Copenhagen, 2200 Copenhagen, Denmark;; ‖NUTRIM, School for Nutrition, Toxicology and Metabolism, Department of Human Biology, Maastricht University Medical Centre, 6200 MD Maastricht, The Netherlands

**Keywords:** Plasma or serum analysis, SWATH-MS, Label-free quantification, Clinical proteomics, Absolute quantification, data-independent acquisition, high throughput, single shot, stable isotope standards

## Abstract

Comprehensive, high throughput analysis of the plasma proteome has the potential to enable holistic analysis of the health state of an individual. Based on our own experience and the evaluation of recent large-scale plasma mass spectrometry (MS) based proteomic studies, we identified two outstanding challenges: slow and delicate nano-flow liquid chromatography (LC) and irreproducibility of identification of data-dependent acquisition (DDA). We determined an optimal solution reducing these limitations with robust capillary-flow data-independent acquisition (DIA) MS. This platform can measure 31 plasma proteomes per day. Using this setup, we acquired a large-scale plasma study of the diet, obesity and genes dietary (DiOGenes) comprising 1508 samples. Proving the robustness, the complete acquisition was achieved on a single analytical column. Totally, 565 proteins (459 identified with two or more peptide sequences) were profiled with 74% data set completeness. On average 408 proteins (5246 peptides) were identified per acquisition (319 proteins in 90% of all acquisitions). The workflow reproducibility was assessed using 34 quality control pools acquired at regular intervals, resulting in 92% data set completeness with CVs for protein measurements of 10.9%.

The profiles of 20 apolipoproteins could be profiled revealing distinct changes. The weight loss and weight maintenance resulted in sustained effects on low-grade inflammation, as well as steroid hormone and lipid metabolism, indicating beneficial effects. Comparison to other large-scale plasma weight loss studies demonstrated high robustness and quality of biomarker candidates identified. Tracking of nonenzymatic glycation indicated a delayed, slight reduction of glycation in the weight maintenance phase. Using stable-isotope-references, we could directly and absolutely quantify 60 proteins in the DIA.

In conclusion, we present herein the first large-scale plasma DIA study and one of the largest clinical research proteomic studies to date. Application of this fast and robust workflow has great potential to advance biomarker discovery in plasma.

## Abstract (precis)

We established a robust capillary-flow data-independent acquisition MS platform capable of measuring 31 plasma proteomes per day without the need of repeated acquisition of the same sample. We acquired 1508 samples of the DiOGenes study (multicentered, Europa-wide caloric restriction weight loss and maintenance study of overweight and obese, non-diabetic participants). This was achieved using a single analytical column. Comprehensive biological reactions to weight loss and maintenance were observed.

## Graphical Abstract

**Figure:**
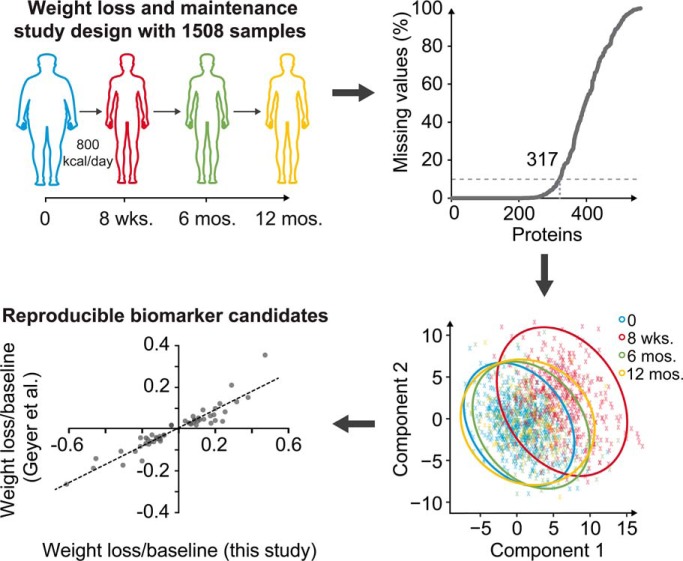

**Highlights**

- Robust capillary-flow DIA was established at 31 clinical samples per day.
- 1508 samples of dietary intervention study DiOGenes were measured on a single column.
- Comprehensive biological reactions to weight loss and maintenance were observed.
- Comparison to independent studies shows high reproducibility of potential biomarkers.

The circulatory system is the primary link between all parts of the body in both humans and animals. As such, it harbors the potential to indicate the health status of any key organ. Blood delivers necessary substances such as nutrients and oxygen to cells and transports metabolic waste products away from those same cells. It is also vital to the immune system-based host defense mechanisms. Blood consists of cells suspended in a liquid called plasma (1). Blood is well suited for diagnoses based on biomarkers, because it is readily obtainable with minimally invasive and repeatable sampling and large biobanks exist for retrospective analyses.

Up to now, 3500 to 5000 proteins have been measured in plasma or serum (2, 3). In addition, there are good arguments that all human proteins might be present in plasma to a certain extent given that blood flows through almost all organs of our body. However, not many more than 100 protein biomarkers from plasma have been cleared/approved by the Food and Drug Administration (FDA)^1^ (4). This contrasts with the high number of protein biomarker studies performed (5). This fact indicates that is difficult to find, validate, and translate new disease or health biomarkers in plasma.

Biomarker discovery has proven to be a complicated, multistage process. Specifically, in proteomics, many biomarker candidates have been generated, but only a few could be validated, and even fewer are used today in the clinic. Potential causes for this are inappropriate study design, limited sample sizes (*e.g.* large interindividual variations were underestimated or technological hurdles to measuring large sample sets) and poor sample quality (5–7). Because of the large individual heterogeneity observed in plasma proteomics (*e.g.* from age, sex, ethnicity and lifestyle), the typical number of samples per condition could be more than a hundred to ensure enough statistical power (8).

A field where these above-mentioned difficulties apply is weight loss studies. Obesity is a medical condition affecting large parts of the population worldwide and is rising rapidly in many places (9, 10). Excess of fat negatively impacts health and can lead to multiple comorbidities including cardiovascular and endocrine diseases such as diabetes, or cancer. It is not fully understood how excess fat manifests its negative effects for the body and how differences occur between individuals (11). Obesity related metabolites are clearly detectable in plasma including fatty acids in lipoproteins and increased chronic inflammation is observed (*e.g.* higher C-reactive protein levels) (12, 13). Obesity can be addressed by prevention (physical activity, nutrition and behavioral changes) and medical treatment but there remains an unmet need for both better treatment options and early diagnosis of obesity's comorbidities. To date, medication that controls appetite has had little success because of adverse side effects and possibly also large inter-person variability in response.

To advance the understanding of obesity, the European-wide weight loss and maintenance study called DiOGenes was initiated (http://www.diogenes-eu.org) (14, 15). The hundreds of participants were overweight or obese people (body mass index (BMI) >27). Eight weeks of weight loss during a low caloric diet (800 kcal·day-1 over 8 weeks) were followed by 6 months of weight maintenance (with an extra six months for the two leading centers). The study was performed in eight locations in Europe. Because of its controlled design and large sample numbers, the DiOGenes study has the potential to lay the foundation for personalized nutrition and dietary intervention.

To perform successful protein biomarker discovery experiments with large enough sample cohorts, mass spectrometry (MS)-based proteomics is in principle, well suited. The technological ability of MS-based proteomics has drastically improved over the last several years (16–19). Currently, the MS biomarker discovery approach has several key features that could render it the preferred platform for protein biomarker discovery, namely its high specificity, protein coverage and the accessibility of PTMs. Historically, MS-based proteomics has not been used in biomarker discovery studies, where large numbers of samples are routinely measured. Recently, several large-scale plasma MS studies were performed by Liu *et al.* (342 proteomes of 342 samples) (20), Cominetti *et al.* (1000 proteomes in 300 TMT experiments of 1000 samples) (21) and Geyer *et al.* (1276 proteomes of 319 samples) (22). Common to all three of these studies is that they used nano-flow liquid chromatography (LC), which generates long injection-to-injection overheads. Cominetti *et al.* performed depletion of abundant plasma proteins with concatenated isobaric labeling experiments, rendering the experiments expensive and sensitive to sample preparation performance (23), whereas less prone to nano-LC instabilities. Additionally, the use of data-dependent acquisition (DDA) mode generates sparse datasets with irreproducible sampling of the peptides in the sample (24), making statistical analysis more difficult and reducing the power of the large sample numbers. To reduce this problem of DDA, multiple re-injections of the same sample were performed (12, 21). From this study, we concluded that two main challenges that remain to be addressed are reproducible MS acquisition and fast, robust chromatography.

The recently established versions of data-independent acquisition (DIA) achieved excellent reproducibility at high coverages making multiple sample re-injections obsolete (24, 25). Additionally, an ∼10-fold higher flow rate LC has been shown to be more robust and reproducible than nano-flow LC (26, 27) and was shown to be compatible with DIA (27, 28). Therefore, we combined higher flow LC with DIA MS in this study.

Herein, we demonstrate the ability to profile 1,508 plasma proteomes from the DiOGenes weight loss and maintenance study with a label-free, single shot DIA and robust capillary flow chromatography approach. At a rate of 31 samples per day, we were able to identify and quantify 565 proteins with high reproducibility, with 459 proteins being identified with two or more peptide sequences per protein (data set completeness of 74%) and on average 408 proteins (5246 peptide sequences) per acquisition. We found proteins and pathways consistently altered after weight loss and during the maintenance phase. Comparison to related and unrelated studies of weight loss clearly demonstrated the general validity of biological findings obtained with large-scale plasma proteomic experiments. Therefore, we anticipate a high impact of the capillary flow DIA large-scale proteomic method in clinical research.

## EXPERIMENTAL PROCEDURES

### 

#### 

##### Ethics

The studies were performed in agreement with the Helsinki declaration and approved by local ethical committees in the respective countries: the Medical Ethics Committee of the University Hospital Maastricht and Maastricht University, the Netherlands (NL); the Committees on Biomedical Research Ethics for the Capital region of Denmark, Denmark (DK); the Suffolk Local Research Ethics Committee, United Kingdom; the University of Crete Ethics Committee, Greece; the Ethics Commission of the University of Potsdam (Germany); the Research Ethics Committee at the University of Navarra, Spain; the Ethical Committee of the Institute of Endocrinology, Czech Republic; and the Ethical Committee to the National Transport Multiprofile Hospital in Sofia, Bulgaria. The Cantonal Ethics Committee for Research on Human Beings, Vaud, Switzerland approved the study protocol to be performed. All study participants signed an informed consent document after verbal and written instructions, and according to local legislation. The Cantonal Ethics Committee for Research on Human Beings, Vaud, Switzerland approved the study protocol to be performed by Biognosys (Schlieren, Switzerland).

##### Study Design

DiOGenes is a multicenter, randomized controlled dietary intervention study, involving eight European countries (15). Briefly, 938 overweight/obese, non-diabetic, adults (BMI between 27 and 45 kg/m^2^, blood fasting glucose below 6.1 mmol/L) underwent an 8-week weight loss phase using a complete meal replacement low calorie diet (LCD). The LCD provided 800 kcal/day (Modifast from Nutrition et Santé, Revel, France). Among the 781 participants who completed the LCD phase, 773 achieved > 8% weight loss and were randomized to a 26-week weight maintenance diet. A total of 548 subjects completed the 6-month weight maintenance diet (WMD) phase. By study design, the two leading intervention centers (DK and NL) continued the WMD for an additional 26-weeks. This design led to a large plasma sample collection (*n* = 1508) available at different clinical intervention day (CID): CID1 corresponding to baseline, CID2 to the LCD termination, CID3 to the 26-week WMD termination and CID4 to the 52-week WMD termination (14).

##### Materials

Iodoacetamide, tris(2-carboxyethyl)phosphine, trifluoroacetic acid, formic acid, ammonium formate, acetonitrile, HPLC water, ammonium bicarbonate, and urea were purchased from SIGMA-Aldrich (Munich, Germany), trypsin sequencing grade from Promega (Madison, WI), PlasmaDeepDive kit from Biognosys.

##### Sample Preparation

1508 human plasma samples were obtained from the European DiOGenes project (14). They were thawed again directly before the preparation for proteomic analysis. The DiOGenes sample set was randomized over 17 96-well plates (supplemental Fig. S1). Each plate contained four pooled plasma samples (twice CID1 and twice CID3 pools) that were prepared together with the other samples. The plasma samples were prepared with the Sample Preparation Kit Pro (Biognosys). In brief, volumes of 10 μl plasma were mixed with 90 μl of denaturation buffer, reduced at 37 °C for 30 min followed by alkylation in the dark for 30 min. Then, 15 μl of denatured plasma were mixed with ammonium bicarbonate buffer and 2 μg of trypsin. Digestion was performed at 37 °C for 3 h and stopped with the digestion stop solution. Desalting was performed using MacroSpin C18 96-well plates (The Nest Group, Southborough, MA) following the manufacturer's instructions. For the DIA analysis, dried samples were resuspended in 50 μl LC of solution A containing iRT and PlasmaDeepDive kits. Finally, the plates were centrifuged at 14,000 × *g* at 4 °C for 30 min, prior to acquisition. The samples were then transferred to 96-well ACQUITY UPLC 700 μl sample plates (Waters, Milford, MA).

##### High pH Reversed Phase Fractionation

For library generation, pooled samples from all samples were fractionated using high pH reversed-phase (HPRP) chromatography. 200 μg of the digest was adjusted to pH 10 using 0.2 m ammonium formate. Next, each sample was fractionated using HPRP separation on a Dionex UHPLC (Thermo Scientific, Waltham, MA) with a 2.1×150 mm ACQUITY UPLC CSH C18 1.7 μm column (Waters) at 60 °C with 0.3 ml/min flow rate and a 30 min gradient with buffer A (20 mm ammonium formate and buffer B acetonitrile, see supplemental Table S1); 1 min micro-fractions were pooled into 15 fractions using fraction pooling.

##### Plasma Capillary Flow DIA Setup Establishment

An Orbitrap Fusion Lumos (Thermo Scientific) was connected to an ACQUITY M-Class (Waters) using Acquity M-Class UPLC CSH 1.7 μm × 15 cm columns (Waters) and Easy Transfer lines (Thermo Scientific) for electrospray (supplemental Fig. S2*A*). The following parameters were identified and optimized as relevant parameters influencing the performance of identification and quantification and robustness: gradient length, column length and diameter, flow, emitter tip type and size, sample amount loaded, electrospray current and transfer tube temperature. Reversed-phase columns of different inner diameters (75, 150, and 300 μm) were tested. All three sized columns performed similarly in terms of identification and quantification performance for the gradient lengths relevant for this study (up to 60 min). Therefore, 300 μm ID columns were chosen because they were more robust when separating plasma samples. Up to date we did not observe any column clogging on the 300 μm ID column in our laboratory and the columns lasted typically >2,000 injections (5 columns, back pressure increase of about 14%). Optimized DIA methods were generated for the different gradient methods as described in (25). A gradient length of 40 min resulted in 97% of the identifications possible on that setup (judged for gradients from 5 to 60 min) (supplemental Fig. S2*B*). A flow rate of 5 μl/min for a gradient of 40 min resulted in 13% overhead from injection to injection (*i.e.* 6 min) (supplemental Fig. S2*C* and S2*D*). A sample load of 5 μg resulted in the maximum number of identified and quantified proteins (supplemental Fig. S2*E*). In summary, this setup enabled the acquisition of 31 samples per day in a reproducible, sensitive and robust manner.

##### Mass Spectrometric Acquisition of the DiOGenes Sample Set

For the capillary flow LC-MS setup, 5 μg of each plasma sample was analyzed using one column setup with an ACQUITY UPLC M-class CSH C18 1.7 μm column of 300 μm×150 mm dimensions (Waters) at 50 °C on an ACQUITY UPLC M-class system (Waters) connected to an Orbitrap Fusion Lumos Tribrid mass spectrometer (Thermo Scientific). A capillary flow Easy-spray transfer line (20 μm) was used with an Easy- Spray source (Thermo Scientific). The peptides were separated with a 40 min segmented gradient (supplemental Table S2). The flow rate was 5 μl/min. The solvents were A: 1% acetonitrile and 0.1% formic acid in water and B: 85% acetonitrile and 0.1% formic acid in water. “Loop” was set to partial with offline after 1.1 min. The seal wash was 90 min and run time was set to 45 min. Following MS parameter were used. The RF lens frequency was set to 40%, the spray voltage was set to 3 kV, the ion transfer tube was set to 300 °C and the internal calibration was used with ion 445.12003 *m*/*z*. For DDA, the quadrupole isolation width was set to 1.6 *m*/*z*. The method cycle time was set to 3 s. The full scan was performed between 350–1650 *m*/*z* at 60 k resolution (AGC target of 1 × 10^6^ or 20 ms injection time). HCD fragmentation was set to normalized collision energy of 27%. The dependent MS2 scans were recorded at 30k resolution with an AGC of 2 × 10^5^ and a max. fill time of 50 ms. The first mass was fixed at 120 *m*/*z*. The intensity threshold was set to 5 × 10^4^, charge states 2–5 were included and the dynamic exclusion was set to 10 s with 25 ppm mass tolerance and isotopes were excluded. The DIA method consisted of a MS1 scan from 350 to 1650 *m*/*z* at 120 k resolution (AGC target of 1 × 10^6^ or 20 ms injection time). Then, 33 DIA segments were acquired at 30 k resolution with an AGC target 1 × 10^6^ and 55 ms for maximal injection time (supplemental Table S3). The setting “inject ions for all available parallelizable time” was enabled. HCD fragmentation was set to normalized collision energy of 27%. The spectra were recorded in profile mode. The default charge state for the MS2 was set to 3.

During the acquisition of the first four plates of the DiOGenes sample set, a failure of the cooling of the MS facility occurred and was not fixed until the end of plate 6. For that reason, two plates were reacquired at the end of the whole sequence. The mass spectrometer was recalibrated after plates 6 and 10. During plate 8, the ion funnel of the Orbitrap Fusion Lumos was cleaned.

##### Mass Spectrometric Data Analysis

To perform Tier 3 analyses (29), DIA spectra were analyzed with Spectronaut Pulsar X 12.0.20491.6 (24). The default settings were used. In brief, retention time prediction type was set to dynamic iRT (adapted variable iRT extraction width for varying iRT precision during the gradient) and correction factor for window 1. Mass calibration was set to local mass calibration. Interference correction on MS1 and MS2 level was enabled. The algorithm is based on correlation of extracted ion currents to a consensus elution profile (24). The false discovery rate (FDR) was estimated with the mProphet approach (30) and set to 1% at peptide precursor level and 1% at protein level (31).

The DDA spectra were analyzed with the MaxQuant (Version 1.6.0.1) software (32) with default settings using the Andromeda search engine (33). Digestion enzyme specificity was set to Trypsin/P. Search criteria included carbamidomethylation of cysteine as a fixed modification, oxidation of methionine and acetyl (protein N terminus) as variable modifications. Allowing up to 2 missed cleavages. The initial mass tolerance for the precursor was 4.5 ppm and for the fragment ions was 20 ppm. The DDA files were searched against the human UniProt fasta database (state 1st July 2017, 42,223 entries) and the Biognosys' iRT peptides fasta database (uploaded to the public repository). For a global post translational modification analysis, we searched the DDA data with MetaMorpheus (V.0.0.297) (34). The default settings were applied, and Hex modifications were added. For targeted glycation DIA analysis, a Maxquant search with the variable modifications of K,R and protein N-terminal glycation by C_6_H_10_O_5_ (162.0528 Da) was executed. Peptides with localization probability <0.75 were removed. The libraries were generated using the library generation functionality of Spectronaut with default settings.

##### Term Usage Definition

When we use the term peptides in this study, we refer to peptide precursors. When we use proteins, we refer to protein groups as determined by the ID picker algorithm (35) and implemented in Spectronaut. Proteins were counted as single hit identification, if they were identified by precursors derived from a single peptide sequence.

##### Experimental Design and Statistical Rationale

For the analysis of intra individual coefficients of variation (CVs), only individuals having been sampled on all 4 CIDs were considered; for the inter individual CV calculation samples from all 4 CIDs, 4 different, random individuals were combined (resulting in a CV based on four samples for both analyses). For batch effect correction we used batch mean centering (36). Statistical pairwise condition comparison analysis on protein level was performed using Welch's *t* test (paired, the null hypothesis is no change, mean μ = 0, two sided, not assuming equal variance). The *p* values were corrected for multiple testing using the *q*-value approach to control the overall FDR (37). Data were interpreted using Qiagen's Ingenuity® Pathway Analysis (IPA®, Qiagen Redwood City, Hilden, Germany) using all identified proteins as the background dataset, Fisher's exact test right-tailed and *p* values were adjusted by the Benjamini-Hochberg approach. Unsupervised clustering was performed using Manhattan distance and Ward's clustering algorithm. To assess differential abundance of PTMs the fold change of the significantly differential abundant PTMs were corrected by the change of the global protein.

## RESULTS

### 

#### 

##### Establishment and Characterization of a Robust Capillary Flow DIA Setup

In order to acquire the proteomes of 1508 plasma samples, we developed a robust and high-performance LC-MS workflow. Higher flow rate LC makes it more robust and results in shorter gradient overheads, therefore we evaluated capillary flow, which promised a balance between performance and robustness. We selected the Orbitrap Fusion Lumos because of its robust ion source and its excellent performance in both DIA and DDA. The ACQUITY M-Class was chosen for its robustness, high flow capabilities and excellent low LC-MS overhead times. After optimization steps (supplemental Fig. S2), we evaluated the capillary flow DIA setup. Therefore, we analyzed ten replicate injections of a plasma pool prepared using an optimized plasma sample preparation protocol (40 min gradient, 5 μl/min and 5 μg injection; see Methods). For the targeted analysis from DIA, we generated a library for the plasma pool based on 15 HPRP fractions using DDA (8,641 peptide sequences from 661 proteins).

**Fig. 1.**
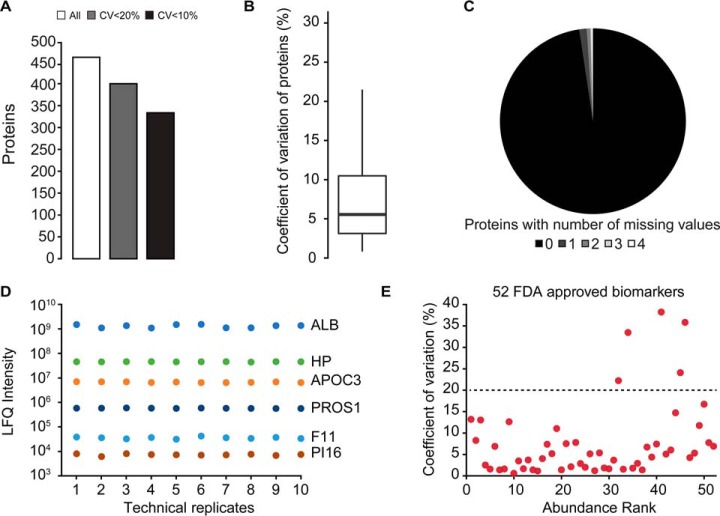
**Capillary flow data-independent acquisition plasma proteomics.** *A*, A plasma pool sample was acquired in ten re-injections on the established capillary flow DIA setup. The total identifications and the identifications with CV below 10% or 20% were calculated. *B*, The CV of each protein was calculated. *C*, The reproducibility of protein identification was analyzed by counting the repeated identification of the individual proteins across the 10 runs. *D*, Quantification reproducibility of six plasma proteins spanning nearly six orders of magnitude (ALB (serum albumin), HP (haptoglobin), APOC3 (apolipoprotein C-III), PROS1 (vitamin K-dependent protein S), F11 (coagulation factor XI), and PI16 (Peptidase inhibitor 16)). *E*, The identification and quantification of FDA approved biomarkers was assessed. 47 of 52 identified biomarkers were quantified with CV below 20% (of a total of 109 described by Anderson *et al.* (38)).

This experiment resulted in the cumulative quantification of 465 proteins (Sept. 2017 Uniprot Fasta with combined, curated immunoglobulins) (Fig. 1*A*). In total, 404/465 (87%) of the proteins were quantified with CVs below 20% in the ten repeated injections. Overall, we observed low CV values (with median CV for all 465 proteins = 5.2% and IQR = 10.7%; see Fig. 1*B*). The reproducibility of identification was very good with less than 1% missing values, and with all 465 proteins consistently quantified in more than two-thirds of the 10 LC-MS runs (Fig. 1*C*). Investigating the list of described plasma proteins, we detected proteins covering nearly 6 orders of magnitude (Fig. 1*D*).

Next, we aimed to assess quantification of well-established biomarkers. Using the 10 technical replicates mentioned above of a pooled plasma sample, we quantified FDA-cleared or FDA-approved protein biomarkers, as listed by Anderson (38). 47 out of the 52 identified FDA-approved biomarkers were quantified with CVs at protein level of less than 20% (Fig. 1*E*).

##### DiOGenes Study Description and Sample Preparation

**Fig. 2.**
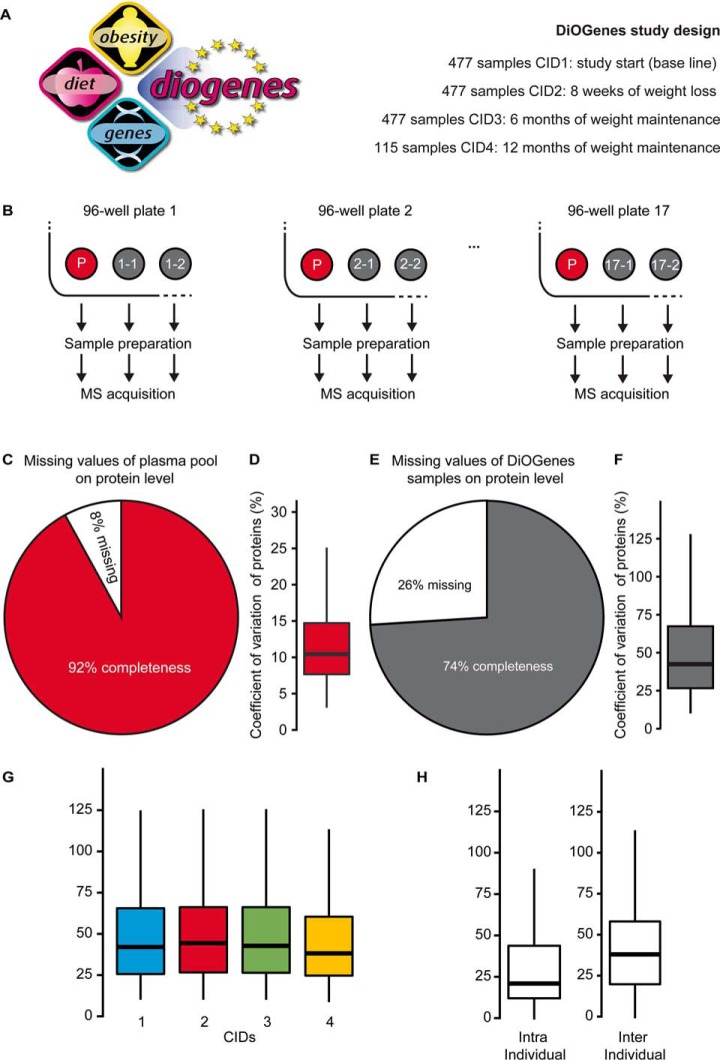
**Exploratory analysis of the DiOGenes dataset.** *A*, Design of the European-wide weight loss and maintenance study called DiOGenes (http://www.diogenes-eu.org). *B*, The 1508 samples and 68 pools were randomized into 17 96-well plates for sample preparation and LC-MS acquisition. *C*, The missing values for the 34 replicates of the CID1-pool were calculated. *D*, The CVs for proteins were calculated for the 34 replicates of the CID1-pool. *E*, The missing values for proteins of the whole sample set of 1474 quality-controlled DiOGenes samples was calculated. *F*, The CVs for proteins were calculated for the full dataset. *G*, The CVs for proteins were calculated for each CID. *H*, For the analysis of intra individual CVs of proteins, only individuals who were sampled at all 4 CIDs were considered and compared with inter individual CVs.

Having optimized and thoroughly tested the capillary flow setup, we started with the acquisition of samples from the multi-centered human dietary intervention study DiOGenes (Fig. 2*A*). Totally, 1576 plasma samples were prepared, comprised of 1508 individual samples and 68 pools. To enable (label-free) absolute quantification, the stable isotope standard (SIS) peptides for 100 plasma proteins of the PlasmaDeepDive kit were added to the digests.

##### Acquisition of the DiOGenes Study of over 1800 Acquisitions on a Single Analytical Reversed Phase Column

The 1576 DiOGenes samples were acquired using the above-described LC-MS setup. Importantly, the whole acquisition sequence was performed on a single analytical column (totally 1803 runs for this manuscript including DDA, DIA and re-injections). The retention time variability for the iRT peptides was low (1.7% CV) and no systematic shift was observed (slope 0.0009). We did not observe a single column blocking in the laboratory. The LC showed some fluctuations during a cooling failure period of the MS-facility and recovered thereafter. Importantly, this had no effect on the acquisition performance as judged by the stable number of protein identifications (supplemental Fig. S3). Intensity, mass calibration and protein identifications per acquisition remained stable over the acquisition sequence. Nonetheless, exploratory, unsupervised clustering analysis of the data set revealed a batch effect between the acquisitions performed before and after the MS-facility cooling failure period (supplemental Fig. S4*A*). Batch mean-centering was performed using the reference pools (supplemental Fig. S4*B*). Because the conditions were evenly randomized over all 96-well plates, no confounding of biological conditions and batches was present.

To be able to retrospectively monitor the performance and reproducibility of the sample preparation and LC-MS acquisition, pooled samples were added into all 17 96-well plates and treated in the same manner as the DiOGenes study samples (Fig. 2*B*). The CID1-pools showed 92% data completeness (pooled samples collected at CID1, two such pools were present on each of the 96-well plates) (Fig. 2*C*); 314 proteins were identified in all pool acquisitions (supplemental Fig. S5*A*) and the CVs for these proteins averaged 10.9% (Fig. 2*D*; 12.9% without batch correction). Similar results were obtained from the CID3-pools (data not shown).

The complete DiOGenes data set was analyzed and 72 LC-MS acquisitions with low protein identifications (below 330 protein identifications) were reinjected. Thereafter, 32 samples were above the threshold. Subsequently, the remaining 40 poor-quality samples were prepared again, resulting in the inclusion of 6 additional plasma samples. Likely, the 31 faulty samples contained no or very diluted plasma in the liquid of the storage tube. Finally, 1542 (*i.e.* 1474 samples and 68 pools) acquisitions were included in the final dataset (2% of samples were excluded, DiOGenes Precursor-level-Spectronaut-Report.zip). The completeness of the full dataset was 74% at the protein level (Fig. 2*E*). Overall, on average 408 proteins were identified (supplemental Fig. S5*B*, and 317 proteins were identified in 90% of the LC-MS DIA acquisitions (supplemental Fig. S5*C*). The average CVs for all label-free protein quantities was 52% across the 1474 samples (Fig. 2*F*), ranging between 48 and 52% within the CIDs (Fig. 2*G*). CVs of the protein quantities of individuals that donated blood samples at all four CIDs were lower than inter-individual CVs (23% CV compared with 38%) (Fig. 2*H*).

##### Stable Isotope Standards in DIA

**Fig. 3.**
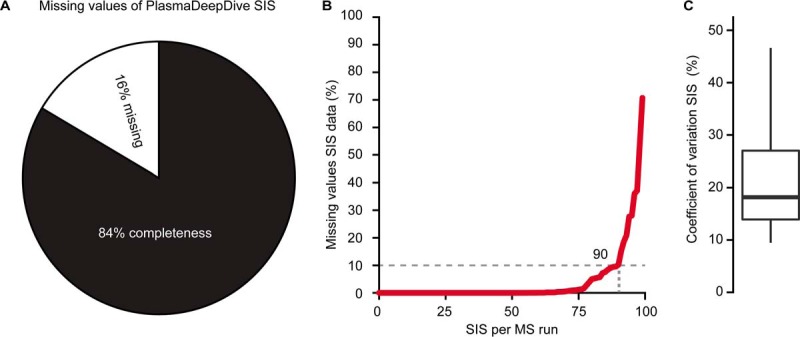
**Stable isotope standard reference peptides for absolute quantification in DIA.** *A*, The missing values in the dataset for the SIS peptides spiked into the 1508 samples of the DiOGenes sample set. *B*, Number of missing values for each SIS reference peptides was calculated. 90 SIS were identified in 90% of the acquisitions. *C*, The CVs for the SIS reference peptides were calculated.

To explore and validate the use of stable isotope standard (SIS) peptides-based quantification, SIS were spiked into the DiOGenes sample set and analyzed in combination with the label-free peptide library. As a result, all the SIS peptides could be detected with 16% missing values (Fig. 3*A*) or 90 of the 99 SIS peptides could be detected in more than 90% of the LC-MS runs (Fig. 3*B*). The median of the CVs for the stable isotope standards was calculated to be 19% (Fig. 3*C*). The endogenous peptides ranged from 10^2^–10^8^ (normalized label-free intensity), the SIS with an intensity in the upper half (10^5^-10^8^) were identified and quantified without missing values (supplemental Fig. S6). Importantly, 68 endogenous counterpart peptides could be detected (73% data set completeness), the use of SIS enabled the identification and quantification of 6% more endogenous peptides counter parts. Importantly, PCA analysis of those absolutely quantified endogenous peptides did not display the previously identified batch effect (supplemental Fig. S7), demonstrating a clear advantage of the isotopic labeling techniques regarding quantification accuracy.

##### Plasma Proteome Characterization

In DiOGenes, the plasma samples were collected in eight countries (centers). Comparing the centers, two centers differed from the rest (supplemental Fig. S8*A*), which was also previously observed (Moreno *et al.*) (13). When we looked at hemolysis judged by hemoglobin contamination of the plasma samples, we saw clear differences between the centers indeed, but no correlation to the centers was observed (supplemental Fig. S8*B* and S8*C*), suggesting an overall low degree of contamination from red blood cells in the plasma samples.

A statistical power analysis showed that for 21% of the proteins a differential abundance of 10% could be detected with ≧ 90% power (433 clinical samples, supplemental Fig. S9). At the baseline (*i.e.* CID1), the proteins with the lowest variability were involved in blood coagulation, whereas the proteins with highest variability were involved in immune response (supplemental Fig. S10), confirming the detection of relevant biological information. Comparing 225 plasma samples from males and 225 plasma samples from females at baseline revealed clear differences between the genders with 207 proteins significantly and differentially abundant. These included the well-described pregnancy zone protein (PZP, 3.6-fold higher in woman) and sex hormone binding globulin (SHBG, 1.6-fold higher in woman), which were among the most significantly differentially abundant proteins. This observation agrees with previous studies (12, 21).

##### DiOGenes Biological Findings

**Fig. 4.**
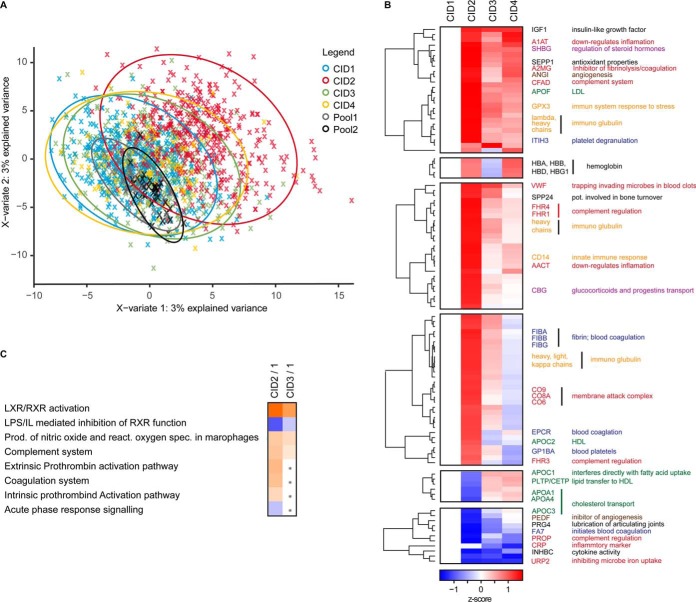
**Biological processes during weight loss and maintenance.** *A*, PLS-DA analysis of the DiOGenes dataset. *B*, The proteins significantly differentially expressed in the dataset were clustered in a heat map. The CID1 time point was set to zero. The annotated protein legend was colored by biological function: (lipid metabolism/transport (green); acute phase response/inflammation (red); blood coagulation (blue); steroid hormone transport/regulation (violet) angiogenesis (brown) and immune system components (yellow). *C*, IPA pathway analysis of the weight loss and weight maintenance to baseline time point, significantly enriched pathways are shown. Orange indicates an activated pathway, white no activity change and blue an inactivated pathway. A dot means no significant enrichment of the pathway.

Clustering of the data showed that the weight loss time point (*i.e.* CID2) differed the most from the baseline (*i.e.* CID1) and also the two weight maintenance time points (*i.e.* CID3 and CID 4) (Fig. 4*A*, supplemental Fig. S11). Using statistical inference of differential abundance, 271 (102 with >10% change) proteins were significantly different between CID1 and CID2, 151 (32 with >10% change) proteins between CID1 and CID3 (Fig. 4*B*, supplemental Fig. S12) and 26 (14 with >10% change) between CID1 and CID4. Eighteen proteins shared altered expression in all time points compared with baseline (DiOGenes Label-free *t* test results.xlsx and DiOGenes Protein Abundance Boxplots.pdf).

All classes of clinically relevant apolipoproteins (20 in total) were profiled in the dataset and all except one responded to the weight loss and showed distinct expression profiles: APOA remained largely unchanged. The levels of a group of apolipoproteins first increased during weight loss and then either increased further (APOD), remained high (APOA5, APOM and APOF) or was reduced to initial levels (APOC2). The levels of the second group were first reduced after weight loss and then either surpassed the baseline levels during weight maintenance (APOH, APOA1, APOA2, APOA4, APOC1, CLUS), went back to initial levels (APOB, APOC3, APOC4) or stayed at reduced levels (APOE, APOL1, SAA1, SAA2, SAA4) (supplemental Fig. S13). Proteins of the membrane attack complex of the complement system were tightly correlated among each other, as were the levels of hemoglobin complex members.

After weight loss, proteins of the acute phase response indicated a reduction in low-grade inflammation (inhibitors were up-regulated and markers were downregulated, *e.g.* CRP, ORM2, SERPINA1 and SAA1 levels dropped and A1AT/AACT increased). This agrees with previous findings in labs and clinics, for example by Esser *et al.* (39). Protein pathway analysis using IPA (40) indicated activated lipid metabolism and increased concentrations of cholesterol and (tri)acylglycerols. It also indicated that accumulation of lipids was reduced while secretion/efflux and transport were activated. Finally, it indicated that the release of prostaglandins was reduced and a reduced recruitment of mononuclear leukocytes (Fig. 4*C*, supplemental Fig. S14).

In the weight maintenance phase, reduced concentrations, secretion and release of fatty acids and lipopolysaccharides were observed based on IPA analysis. Additionally, low grade inflammation remained reduced (Fig. 4*B*, supplemental Fig. S14). Eighteen proteins remained significantly differentially abundant from baseline to the two weight maintenance time points. They are involved in the complement system, the chronic inflammatory process of atherosclerosis (supplemental Fig. S15). Specifically, [1] the adipokine adiponectin remained elevated in weight maintenance, it is involved in the control of fat metabolism and insulin sensitivity (41); [2] apolipoprotein F remained elevated, it is an important regulator of cholesterol transport, (42); [3] apolipoprotein C-II remained below baseline, it plays an important role in lipoprotein metabolism as an activator of lipoprotein lipase and is a component of chylomicrons, very low-density lipoproteins, low-density lipoproteins and high-density lipoproteins (43); [4] vascular cell adhesion protein 1, which may play a pathophysiologic role both in immune responses and in leukocyte emigration to sites of inflammation (44); [5] complement factor H and properdin, which are regulators of the alternative pathway of the complement system (45); [6] sex hormone binding globulin, which regulates the plasma metabolic clearance rate of steroid hormones by controlling their plasma concentration (46); [7] glutathione peroxidase 3, which protects cells and enzymes from oxidative damage and for which a link to obesity was found (47, 48); [8] selenoprotein P, which is involved in the extracellular antioxidant defense properties of selenium and linked metabolic diseases (49); [9] cartilage acidic protein 1, which is a glycosylated extracellular matrix protein and linked to colorectal cancer (50); [10] zinc-alpha-2-glycoprotein increased over time, which stimulates lipid degradation in adipocytes (51) and is a candidate therapeutic agent against obesity via induction of a brown fat-like phenotype in white adipocytes (52, 53).

We analyzed the proteins that significantly changed in quantity between baseline and weight loss separately for males and females (167 individuals each) and discovered an overlap of only 64 proteins. The overlapping proteins showed the same direction of regulation (supplemental Fig. S16). Still, many proteins were significant in either females or males, showing the importance and relevance of a gender-specific approach in dietary interventions and further tailored personalized nutrition.

##### Consistency with Other Large-scale Proteomics Weight Loss Studies

**Fig. 5.**
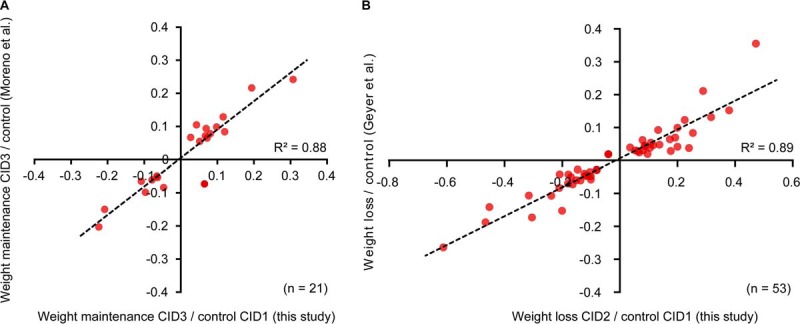
**Comparison of biological findings to related large-scale proteomic weight loss maintenance studies.** *A*, The significantly differentially abundant proteins in this study resulting from the CID3 to CID1 comparison (151 candidate proteins) were compared with the candidate list published by Moreno *et al.* (13) (32 candidate proteins) using the same DiOGenes samples of CID1 and CID3, but analyzing them with the ASAP^2^ pipeline including depletion, isobaric labeling, and DDA acquisition with an Orbitrap Elite mass spectrometer. *B*, Comparison of the significantly differentially abundant proteins between CID2 and CID1 resulting from this study (from 271 candidate proteins) to the results from the independent weight loss study by Geyer *et al.* (22) with the same layout of 8 weeks of weight loss (from 68 candidate proteins), acquired with a Q Exactive HF mass spectrometer.

The DiOGenes plasma samples collected at CID1 and CID3 were previously analyzed using the so-called ASAP^2^ pipeline (21) and interpreted by Moreno *et al.* (13). In their work, depletion of the 14 most abundant plasma proteins was performed, followed by isobaric labeling for sample multiplexing and DDA acquisition in duplicates using a nano-flow LC setup with 150 min gradient separation. Comparing this previous report to the present study, 272 proteins were commonly identified. The results of the statistical comparison of plasma protein changes between CID1 and CID3 were also compared between both studies (Fig. 5*A*). Remarkably, 21 proteins identified to be significantly differentially abundant by Moreno *et al.* (from a total of 38 proteins of which three were not detected in this study) were also found to be significantly differentially abundant with same directionality of change in our study (except two with low fold changes below 10%). In Fig. 5*A*, the slope of the curve was 1.03 (R^2^ = 0.8798), confirming agreement in the quantitative determination of proteins and good performances of MS-based discovery proteomics (note that plasma sample proteomic measurements were separated by about three years). When looking only at the proteins that changed by more than 10%, all commonly identified proteins were significantly changed in both studies. Insulin-like growth factor binding protein 2 (IBP2) was not detected as significantly changed in our study, but insulin-like growth factor 1 (IGF1) was, to which it binds with high affinity (54).

Additionally, we compared the proteomic results of our study to an unrelated weight loss study analyzed by Geyer *et al.* (22). There, the same 8 weeks of caloric restriction was performed. Importantly, when comparing significant differential abundances between baseline (*e.g.* CID1 in DiOGenes) and weight loss (*e.g.* CID2 in DiOGenes), a large overlap of 53 proteins was found and the directions of change were conserved (Fig. 5*B*). Here, the slope of the scatter plot was 0.44, possibly because of the different samples analyzed in the studies, but an R^2^ of 0.89 confirmed the very strong agreement and robustness of biological findings.

##### Glycation Changes of Plasma Proteins

Using DIA, it is possible to interrogate the raw data with different foci. Therefore, DDA of the plasma pools were searched for post translational modifications of peptides (see methods). This revealed a multitude of modifications (see DiOGenes Plasma Modifications.xlsx). We decided to focus non-enzymatic glycation of proteins by glucose which is biologically relevant in the context of obesity and type 2 diabetes. The global PTM analysis indicated that multiple amino acids were glycated. We searched for *N*-linked glycation on lysine, arginine and the protein N terminus (55) using a classical database search. This resulted in a library containing 242 glycation sites on 70 proteins (listed in DiOGenes Plasma Glycation Sites.xlsx). 28 of these proteins were previously reported by Zhang *et al.* (55). The most glycation sites were detected on serum albumin (36 sites), serotransferrin (13) and immunoglobulin kappa constant region (7).

**Fig. 6.**
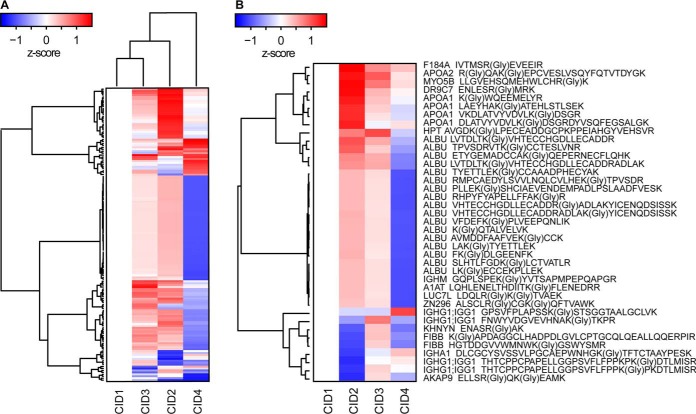
**Non-enzymatic glycation analysis of plasma proteins.** A library was generated containing high quality glycation sites and was then applied to the targeted analysis of the DIA DiOGenes dataset. *A*, All identified glycation sites changes were calculated comparing to CID1 and corrected by the protein abundance change. Subsequently, the data was visualized in a clustered heat map (HC, average linkage). *B*, The glycation sites significantly differentially abundant in the dataset were processed as described above.

Applying this library to the DiOGenes DIA resulted in the identification and quantification of 234 glycation sites. Surprisingly, global unsupervised clustering analysis revealed that CID4 clustered away from the first three other CIDs (Fig. 6*A*). Interestingly, this contrasts with the clustering based on whole protein expression. 16 proteins showed significantly altered states for glycation in the dataset (Fig. 6*B*, see file DiOGenes Plasma Glycation *t* test Result.xlsx). All glycation sites on albumin were slightly elevated after the weight loss period.

## DISCUSSION

We established a robust and sensitive MS proteomic workflow based on capillary flow, single-shot DIA, applicable to large-scale clinical research studies. We profiled a clinical sample set of 1508 samples of the weight loss and maintenance study DiOGenes at a depth of 565 proteins. This is enough to cover the clinically relevant lipoproteins (20 in total) and markers of inflammation, as well as many other important functional blood proteins. The entire workflow demonstrated the high consistency demonstrated by control pools with high reproducibility (92% completeness) and low variance (10% CV) spread across the 1576 samples (Fig. 2). For 32 of the 72 samples with low protein identifications, a reinjection increased the number of identifications above the quality threshold (*i.e.* ≥330 proteins). No “cross reactivity” exists in MS, like with antibodies-based approaches and no MS1 alignment had to be performed. This enabled detection of significant changes of protein levels below 10%. Hence, this resulted in more proteins being detected as significantly, differentially abundant as compared with similar studies (13, 22).

A large-scale proteomics experiment with hundreds of samples needs proper planning. These include sample storage and handling, *e.g.* for randomization of samples or introduction of quality control pools to track the performance of the proteomic workflow (at both sample preparation and LC-MS acquisition levels). The sample preparation and LC-MS were controlled with pools on each of the 96-well plates. The data analysis requires a software able to process and analyze the whole project to ensure proper FDR control, protein quantities and normalization. Statistical testing also needs to be selected correctly to address potential confounding biological variables (*e.g.* more females in a condition, age differences or effects of sample collection at multiple centers).

The wide-ranging differences that were detected in the female and male human plasma proteomes at baseline demonstrate the importance of considering these observations in a plasma proteomics experiment and considering those potential confounders. It can also serve to control the quality of the data using gender, as is regularly performed in the field of genomics (56). Additionally, effects from collection centers (here, in different countries) including genetic background, different diets, environment and blood drawing effects could confound biologically relevant findings. Additionally, protein variance across the study was smaller within an individual than between individuals (Fig. 2*D*), a finding that has been previously observed (22). The sampling of individuals at multiple points of intervention enabled paired statistical tests with the use of unequal cohorts from different centers/genders, keeping statistical power high.

Weight loss and maintenance triggered multiple changes in biological pathways including sustained reduction of low-grade inflammation and the activation of lipid metabolism. These findings were confirmed in an independent weight loss study (22). The sustained reduction of apolipoprotein E demonstrated a positive effect on cardiovascular risk (57).

An unbiased search for PTM modifications revealed a multitude of variations. DIA enabled the comprehensive profiling of 234 glycation sites, for the first time, without the need for enrichment in the samples for glycated moieties. Globally, glycation was observed to increase first, during weight loss, to then drop during the weight maintenance phase to levels below those at baseline. There appears to be a delay in the glycation change post-weight loss. This reduction indicates potential beneficial effects, because glycated proteins are known to trigger an immune system response (58). A glycated protein is considered by the immunological system as an “undesired” species, and consequently its enzymatic digestion is activated (58) *e.g.* glycated albumin in diabetes (59).

The size of the data set also enabled analysis of subgroups and differences, *e.g.* the number of significantly differentially abundant proteins after weight loss was different for men and women. Similar analyses can be performed for centers, diets, ages and BMIs in future investigations.

The robustness of the findings from large-scale proteomics experiments with plasma could be clearly demonstrated in two independent ways by comparison: (1) to the works of Cominetti *et al.* (21) and Moreno *et al.* (13) which used the same plasma samples from the DiOGenes study, but performed depletion and isobaric labeling before MS acquisition and (2) to the results of the analysis of another weight loss study (60), acquired with label-free DDA by Geyer *et al.* (22). These comparisons show that the results of large-scale proteomics experiments are technically and biologically reproducible and can be generalized (Fig. 5).

In contrast to Cominetti *et al.*, in this study no depletion and labeling costs were accumulated and no repeated injections were necessary. The time spent per sample in this study was half that spent by Cominetti *et al.* (Cominetti *et al.* used an older generation Orbitrap Elite Hybrid Ion Trap Orbitrap, whereas we used an Orbitrap Fusion Lumos Tribrid mass spectrometer) and 5.2 times less than Geyer *et al.* (Geyer *et al.* used a Q Exactive HF Quadrupole Orbitrap). (supplemental Table S4). Because of the higher coverage and/or reproducibility, this study was more sensitive in identifying differential abundances (*e.g.* 2.9-fold more for CID3/CID1 than Moreno *et al.* and 60% more than Geyer *et al.* (for weight loss/baseline) at equal LC-MS time.

SIS-based absolute quantification enables additional post-analysis strategies relying on absolute quantities (pathway models, kinetic models, among others). We showed, that SIS are reproducibly identified, if the SIS are spiked in at an intensity corresponding to the upper half of the label-free intensity range of plasma (Fig. 3). Importantly, batch effects generated by the LC and MS did not propagate into the directly, quantified proteins by SIS. Therefore, quantifying the absolute amount of most proteins has the potential to improve the homogeneity of large extended projects and the ability to compare different projects. This is reasonable, because in plasma 300 to 1000 proteins are routinely detected. To reduce batch effects arising from differential sample preparation, addition of known amounts of labeled proteins to native plasma would be the ideal solution (61).

Based on the herein demonstrated efficiency of the large-scale proteomic workflow and the recent evolution of large-scale plasma proteomics, we conclude that the tools are now in place to engage in biomarker discovery. This opens the use of approaches such as capillary flow DIA or the recently introduced Evosep liquid chromatographic system (62) to boost the discovery of new biomarkers, a prerequisite in personalized medicine and nutrition.

## DATA AVAILABILITY

The raw MS data, the spectral libraries and the quantitative data tables have been deposited to the ProteomeXchange Consortium via the PRIDE (63) partner repository with the dataset identifier PXD013231. The projects from Spectronaut can be viewed with the Spectronaut Viewer (www.biognosys.com/spectronaut-viewer).

## Supplementary Material

Supplemental Information

Protein t-test results

Glycation modification in plasma

Glycation t-test results

Post translational modifications of plasma

## Footnotes

^1^ The abbreviations used are:

FDA: Food and Drug Administration
CV: coefficient of variation
DDA: data-dependent acquisition
DIA: data-independent acquisition
FDR: false discovery rate
MS1: peptide precursor survey scan
MS2: fragment ion scan
CID: clinical investigation day
LCD: low-calorie diet
WMD: weight maintenance diet
SIS: stable isotope standard
LC: liquid chromatography
RF: radio frequency
MS: mass spectrometry
PTM: post-translational Modification.
