# Supplementary material for: Analysis of 1508 Plasma Samples by Capillary-Flow Data-Independent Acquisition Profiles Proteomics of Weight Loss and Maintenance
Source: Mol Cell Proteomics. 2019 Apr 4;18(6):1242–54. doi: 10.1074/mcp.RA118.001288 (PMC6553938; doi:10.1074/mcp.RA118.001288)
Supplement: Supplemental Information [file 142681_1_supp_305431_psxs3g.pdf]

Supplemental Material:

Analysis of 1,576 plasma samples of the DiOGenes study - Robust, single shot capillary flow data-independent acquisition to decipher proteomic profiles of weight loss and maintenance

Supplemental Tables:

Supplemental Table 1: HPRP Chromatographic gradient.

min	%B	Gradient
0	1	5
3	6	5
4	8	5
6	10	5
8	12	5
10	13	5
11	15	5
13	16	5
14	18	5
16	19	5
18	20	5
20	22	5
22	24	5
24	26	5
26	28	5
30	39	5
30.1	90	5
31	90	5
31.1	1	5

Supplemental Table 2: LC-MS Chromatographic gradient.

min	%B
1.1	1
3	6
5	9
11	14
17	19
23	24
30	30
41	51
41.05	99
43.05	99
43.1	1
45	1
180	1

Supplemental Table 3: DIA segment distribution.

center (m/z)	Isolation Window (m/z)
361.875	23.75
383.5	20.5
402	17.5
419	17.5
435.5	16.5
451.5	16.5
467	15.5
482	15.5
496.5	14.5
511	15.5
526	15.5
540.5	14.5
555	15.5
570	15.5
585	15.5
600.5	16.5
616	15.5
631.5	16.5
648	17.5
665.5	18.5
683.5	18.5
702	19.5
722	21.5
743.5	22.5
766.5	24.5
791	25.5
818.5	30.5
850	33.5
886	39.5
930.5	50.5
989.5	68.5
1079	111.5
1392.125	515.75

Supplemental Table 4

Comparison of the time spent per sample of different large-scale proteomic studies.

Study	Repeated injections	Multiplexed	Injection to injectio	Time spent per sample (min	Comments
Geyer et al.	4	1	60	240	quadruplicates
Cominetti et al.	2	4	180	90	isobaric labelling
This study	1	1	46	46	

Capillary Flow DIA on DiOGenes study

Supplemental Figures:

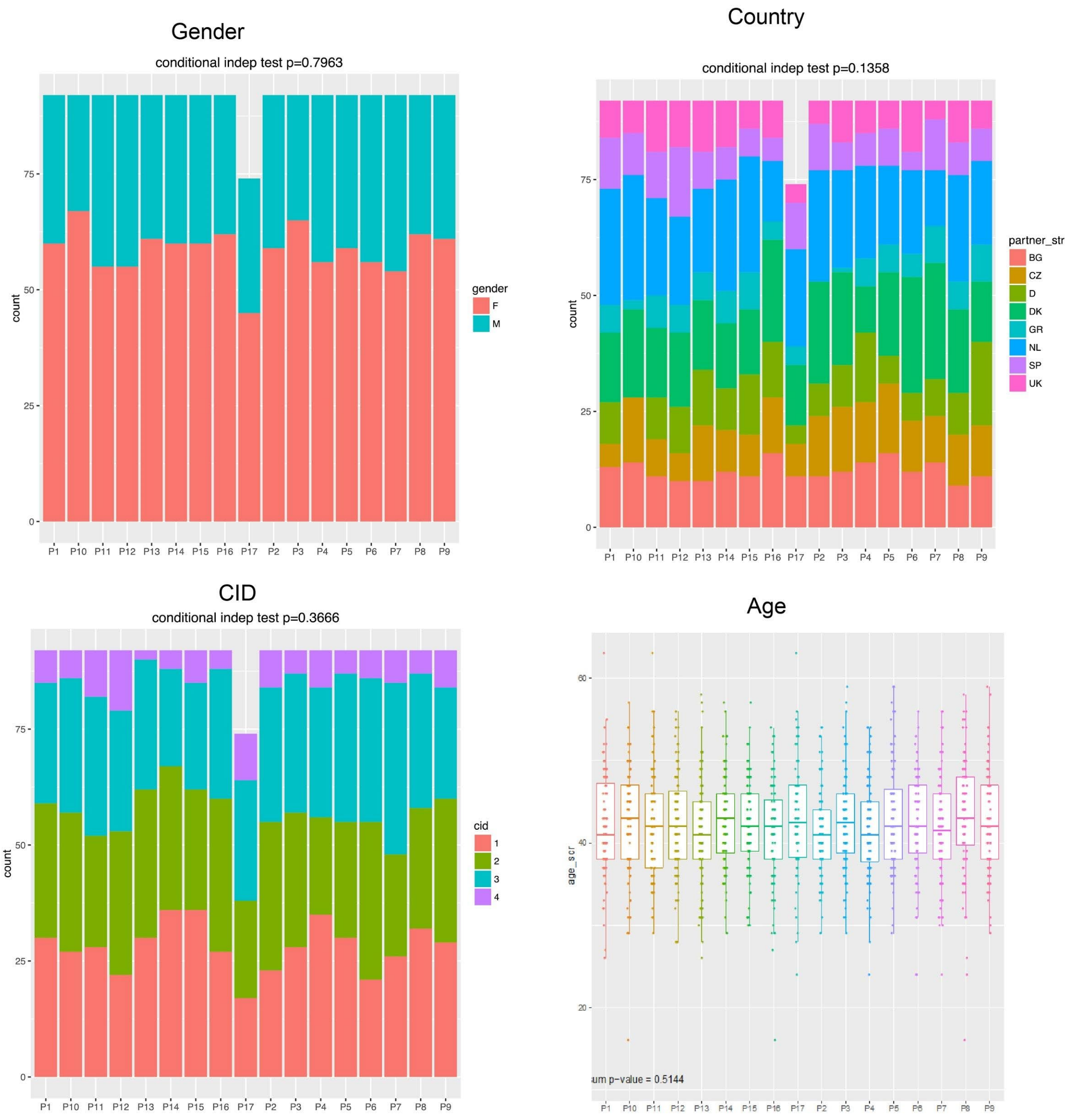

Supplemental Fig. 1: Randomization of sample set.

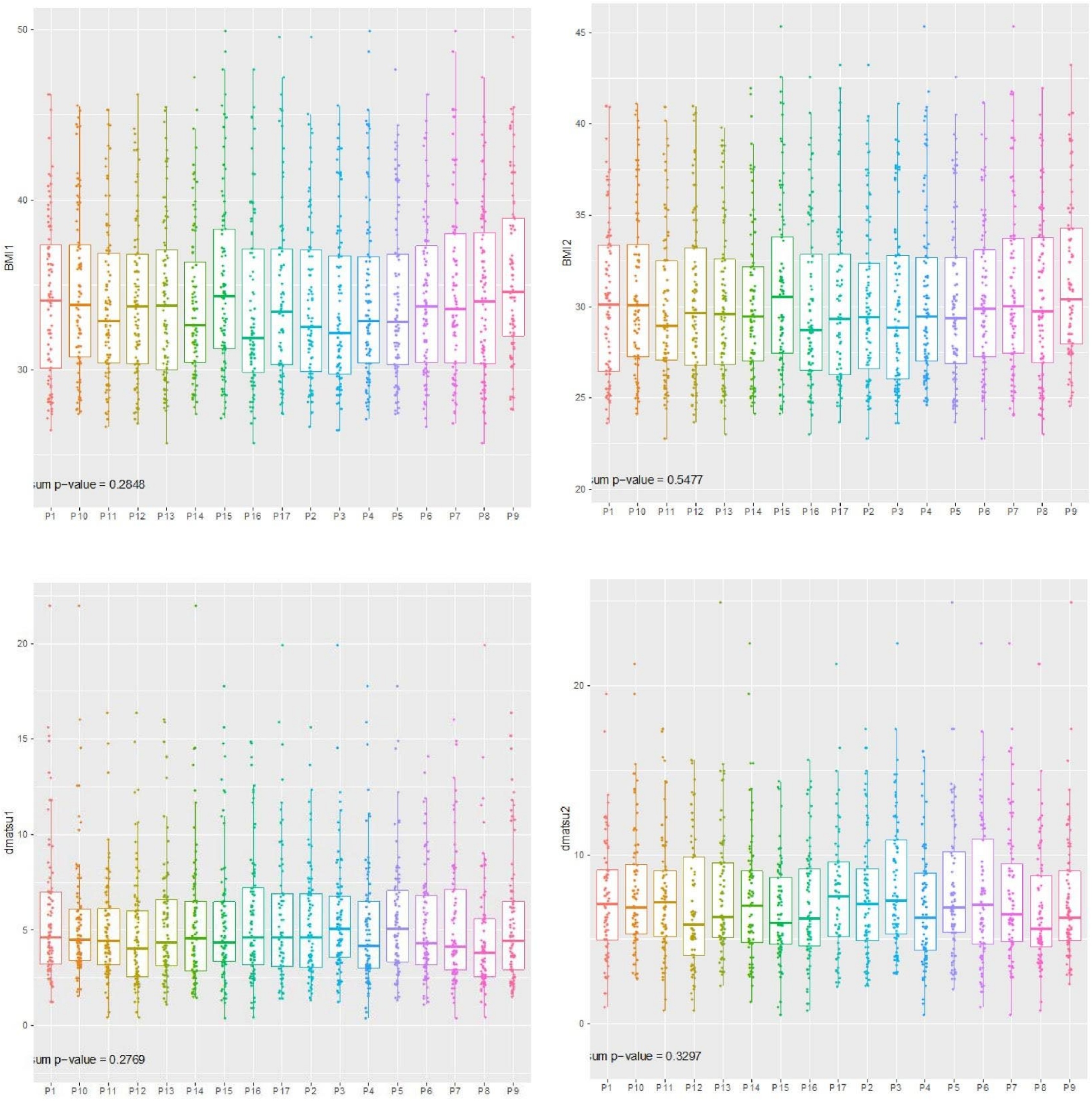

Supplemental Fig. 1: Randomization of sample set.

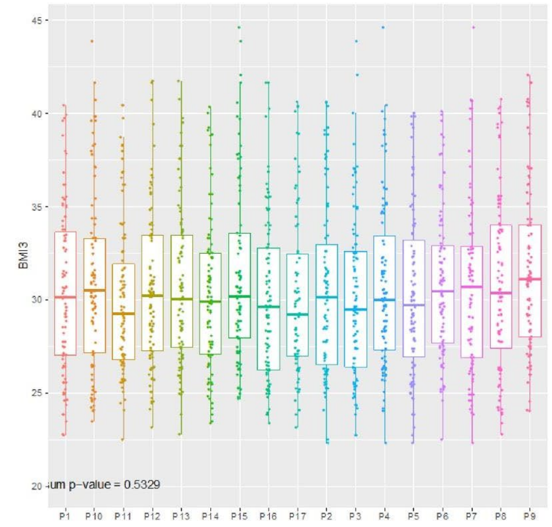

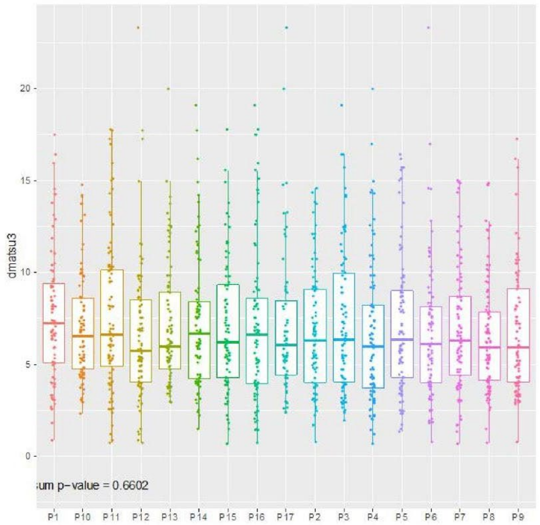

Supplemental Fig. 1: Randomization of sample set.

The DiOGenes sample set was randomized onto 17 96-well plates including four pools on each plate. The randomization was controlled for spontaneous generation of clusters for clinical variables (i.e., gender, country, clinical investigation day, age and BMI). No significant enrichment of a variable on a plate was observed as judged by a multivariate nonparametric test of independence.

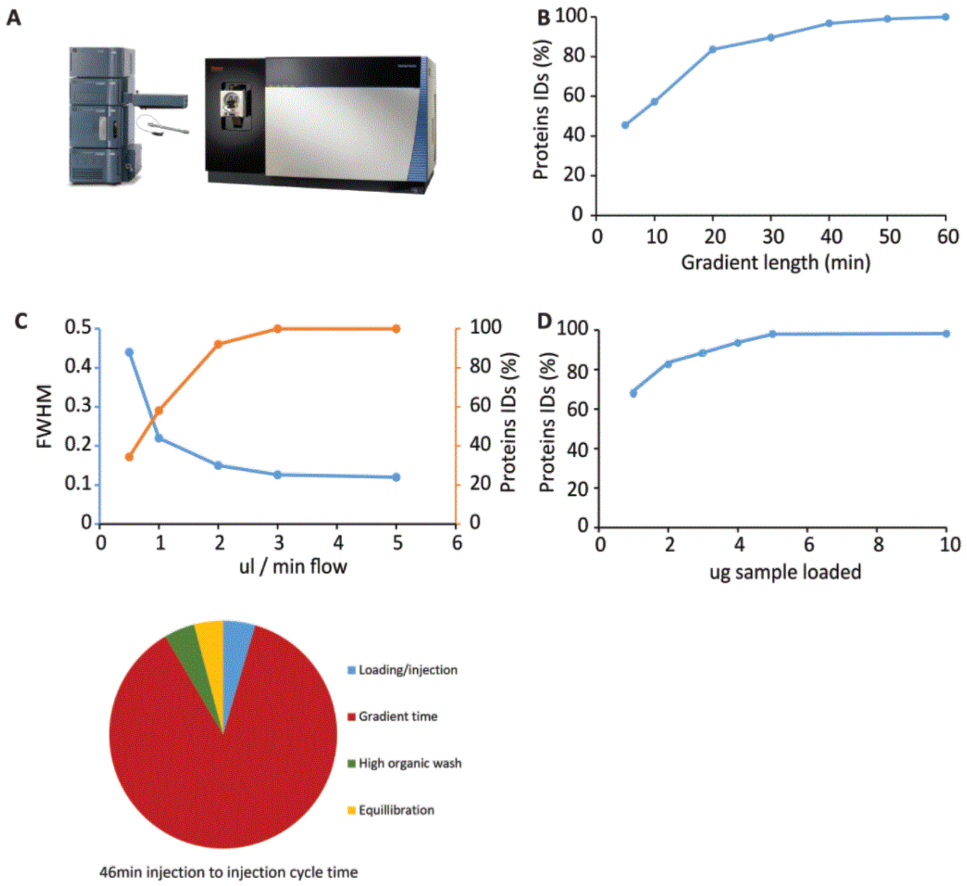

Supplemental Fig. 2: Establishment of a capillary flow setup.

(A) A Waters M-Class UPLC was connected to a Thermo Scientific Orbitrap Fusion Lumos mass spectrometer. A 300 µm*15 cm CSH C18 column 1.7 µm (Waters) was selected. (B) The protein identifications at different gradient lengths were used. (C) The peak capacity in dependence of the flow rate was determined. (D) The protein identifications in dependence of sample loading were evaluated. (E) Visualization of the time spent in an injection-to-injection cycle (46 min LC run time).

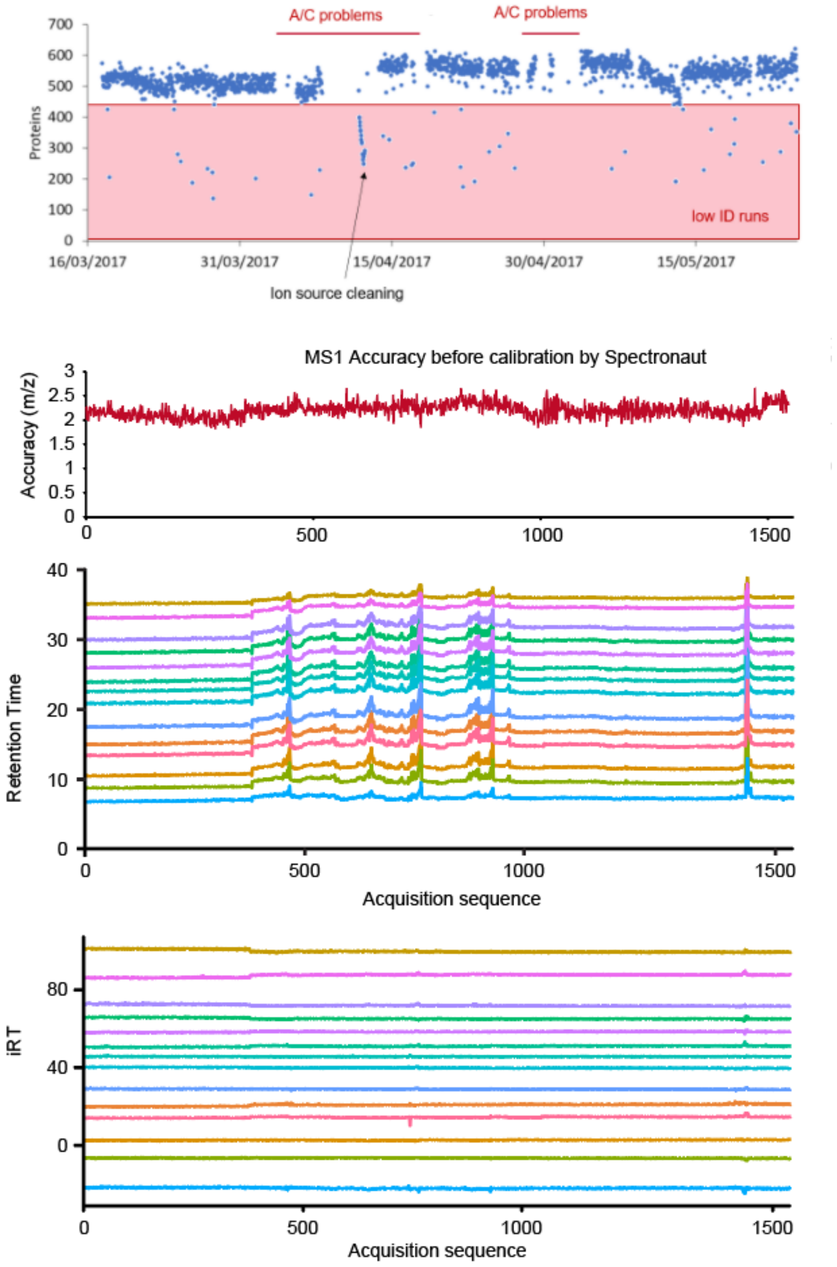

Supplemental Fig. 3: Exploratory analysis of the DIA of the DiOGenes samples.

(A) The proteins were counted per DIA run and plotted on a time axis (no protein FDR). A/C cooling failure of the MS-facility phases were indicated and only cleaning of an internal part of the mass spectrometer (besides the regular transfer tube exchanges). (B) *m/z* mass deviation of the 1542 DIA raw files before re-calibration with Spectronaut Pulsar X. (C) The iRT peptide standards were plotted for the 1542 DIA analyses. During the A/C cooling failure of the MS facility, larger fluctuations in retention times were

observed. (D) Transfer of the retention times into the dimension less iRT scale was performed for the 1542 DIA analyses. This transformation removed the fluctuations generated by the cooling failure.

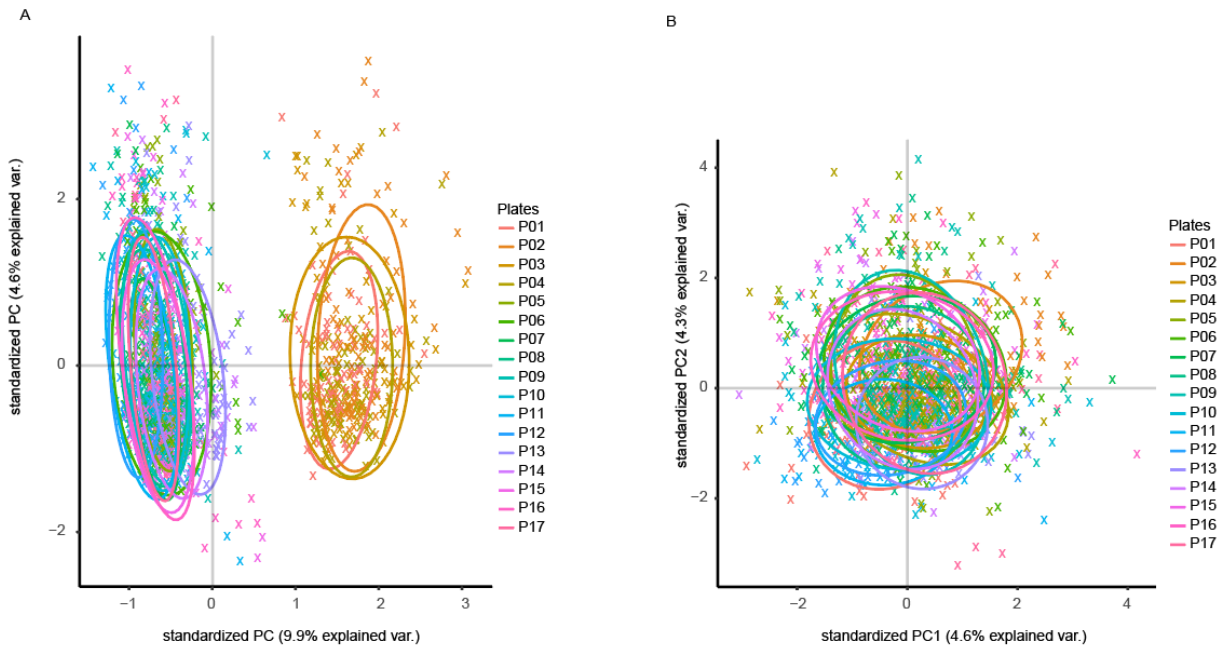

Supplemental Fig. 4: Bach effect analysis and correction.

(A) PCA analysis of the DIA targeted analysis from the DiOGenes dataset was performed. A distinct batch effect between plate 4 and 5 was apparent. This correlated with the cooling failure of the MS-facility. (B) Protein wise mean batch correction was applied to the DIA dataset and a PCA was performed again.

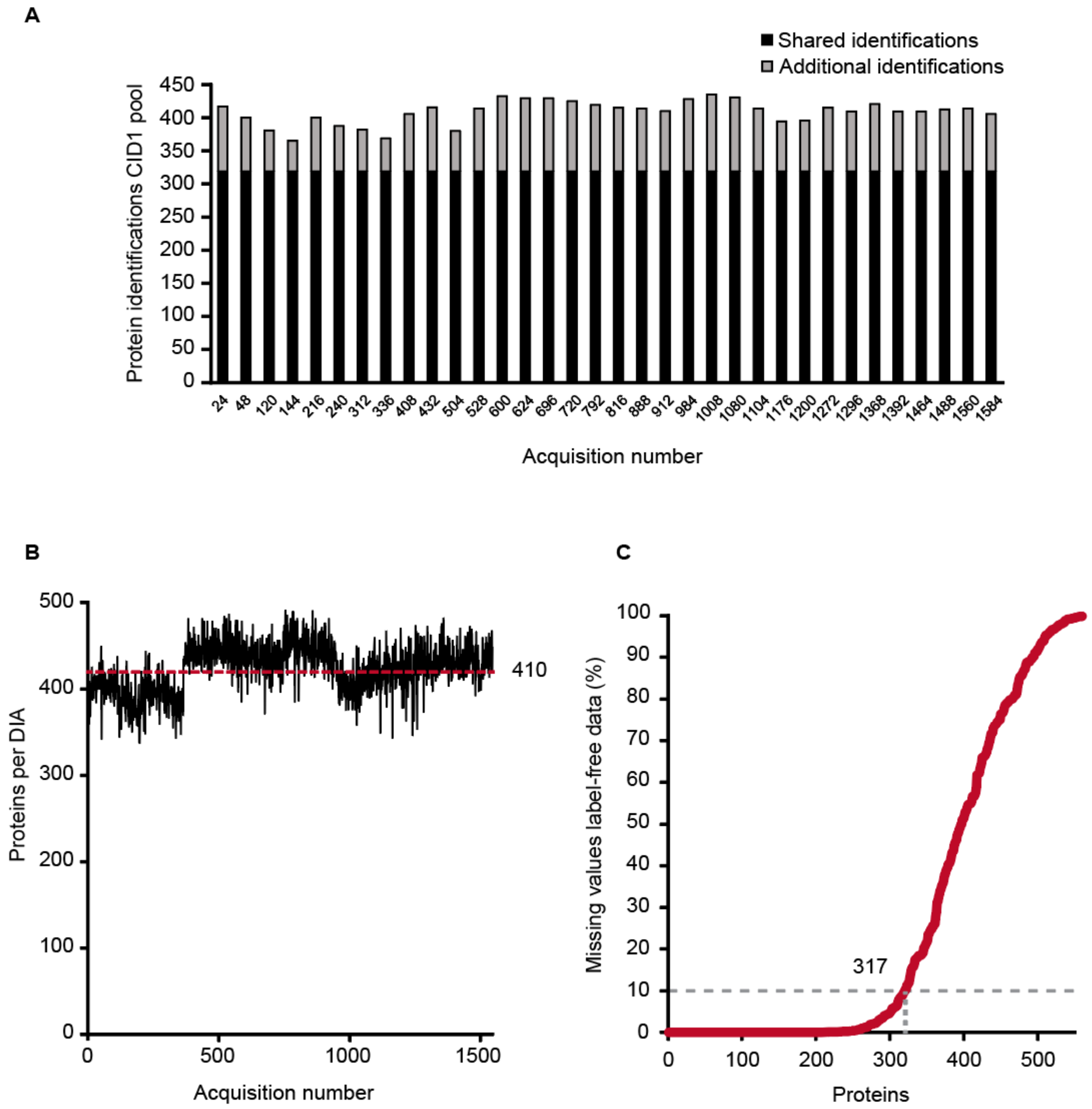

Supplemental Fig. 5: Proteome coverages and data completeness.

(A) Identifications were calculated for the 34 workflow replicates of pool of the CID1 samples. (B) Identifications were calculated for the 1542 plasma proteomes included in the final dataset. On average 410 proteins were identified per sample (red line). (C) The missing values per protein were counted. 317 proteins were identified in 90% of the samples.

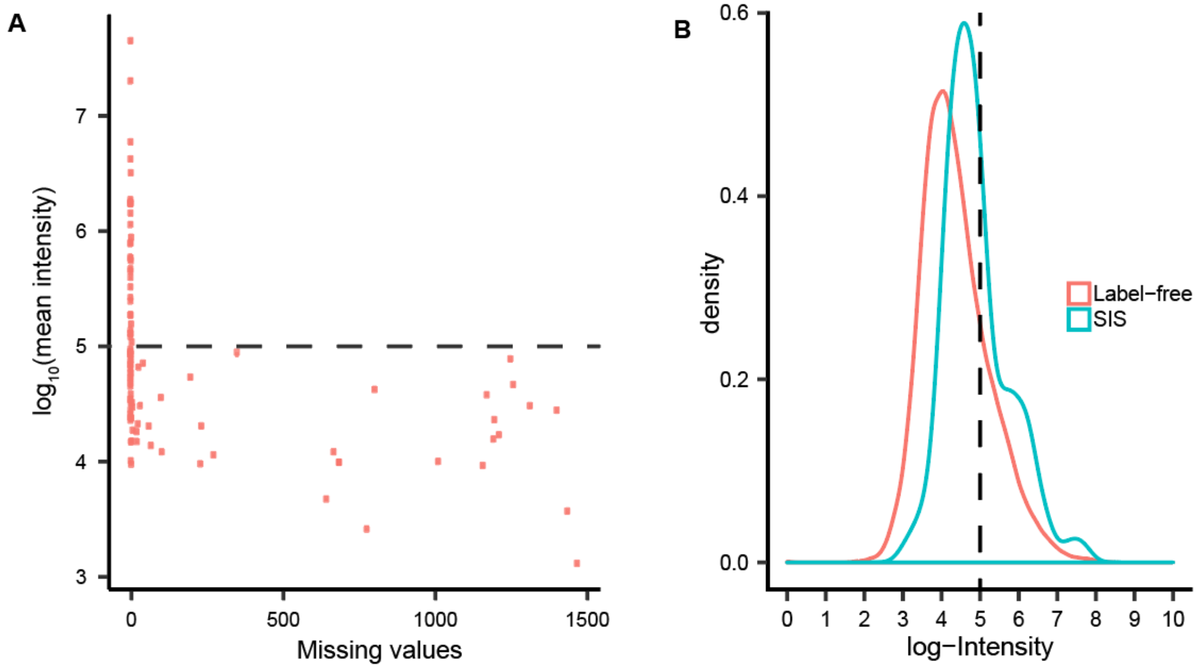

Supplemental Fig. 6: Reproducibility of SIS identification:

(A) The mean SIS intensity and the missing values in identification were calculated. (B) The distribution of the label-free intensities and the SIS intensities were calculated. The dotted lines represent the threshold of 10^5, above which all SIS was identified without missing values. The log base is 10.

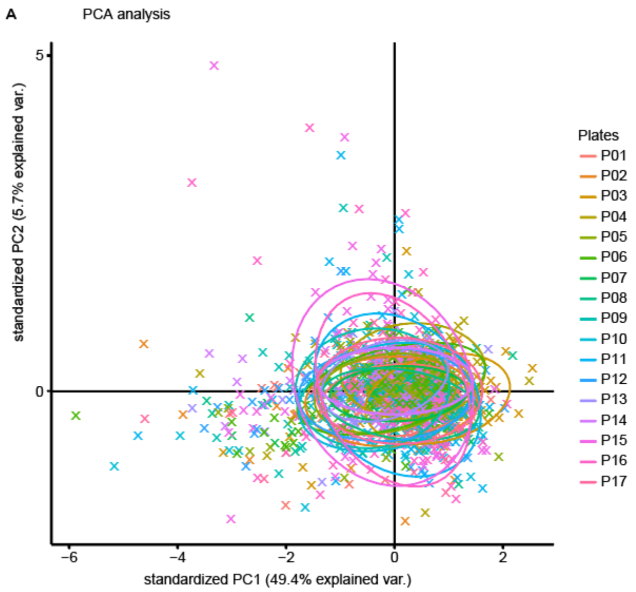

Supplemental Fig. 7: SIS analysis batch effect analysis:

PCA analysis of directly absolutely quantified proteins via SIS reference peptides of the DiOGenes. No batch effect was observed between the 96-well plates.

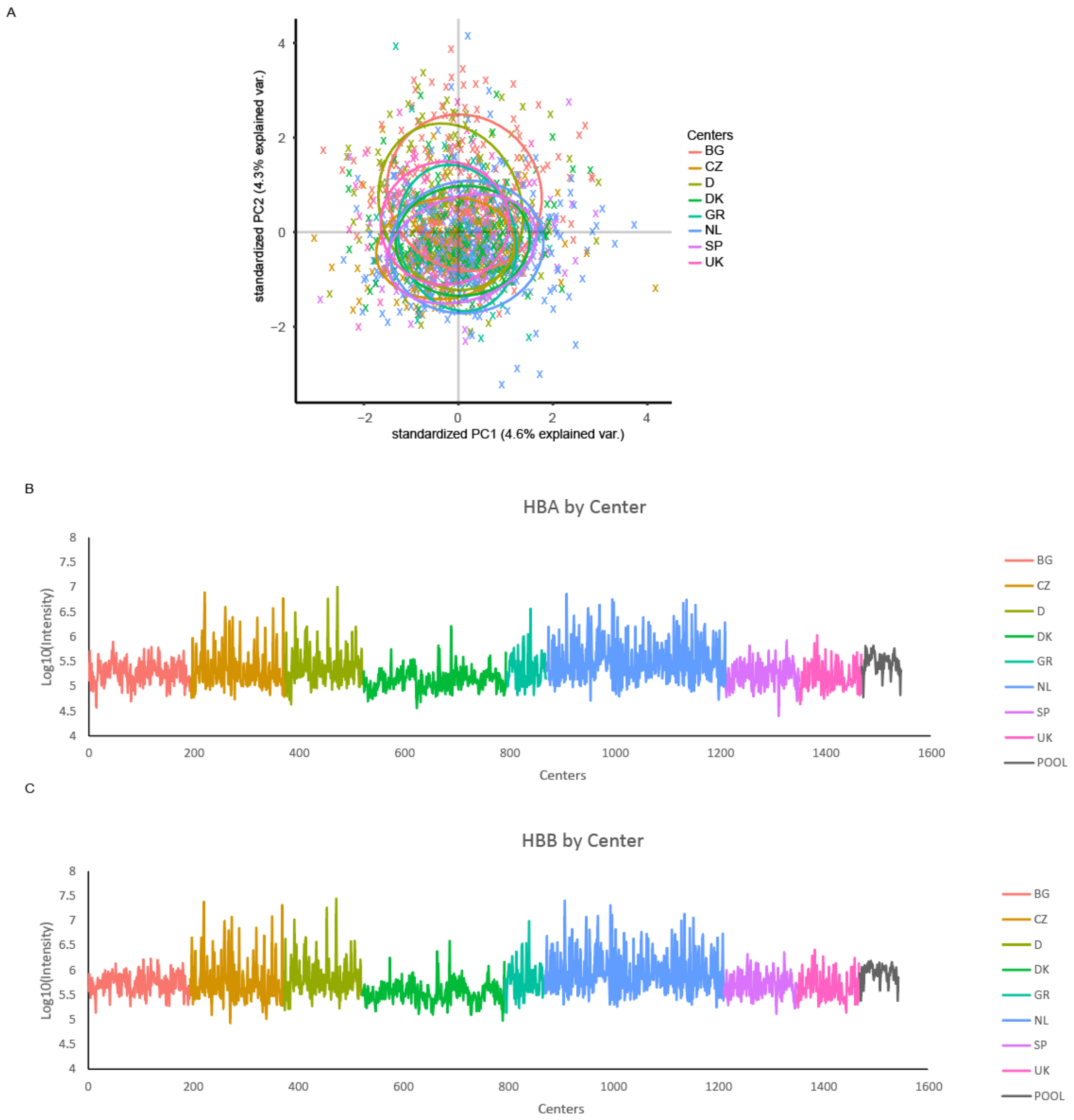

Supplemental Fig. 8: Center effect of DiOGenes sample collection.

(A) PCA analysis of the 1542 DIA of the DiOGenes sample set with annotation of the countries, where the samples were collected. (B) Hemoglobin alpha chain analysis organized by country of sample collection and acquisition order. (C) Hemoglobin beta chain analysis organized by country of sample collection and acquisition order.

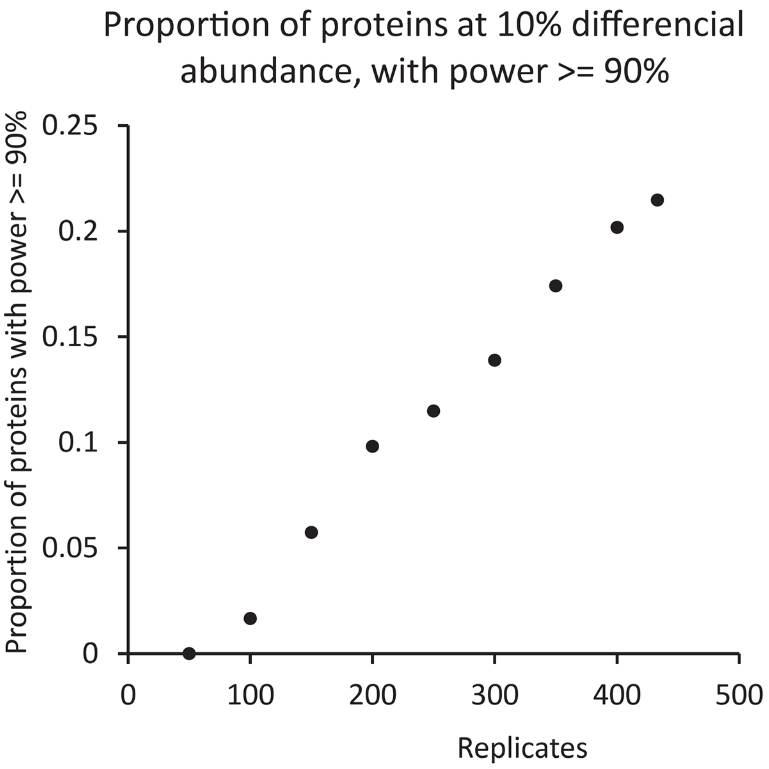

Supplemental Fig. 9: Analysis of detection of 10% change at statistical power of 90% at baseline (standard deviation at baseline, multiple testing correction by the Bonferroni method, ssize package Ver. 1.56.0 for R). The number of clinical plasma samples were ramped from 50 to 433.

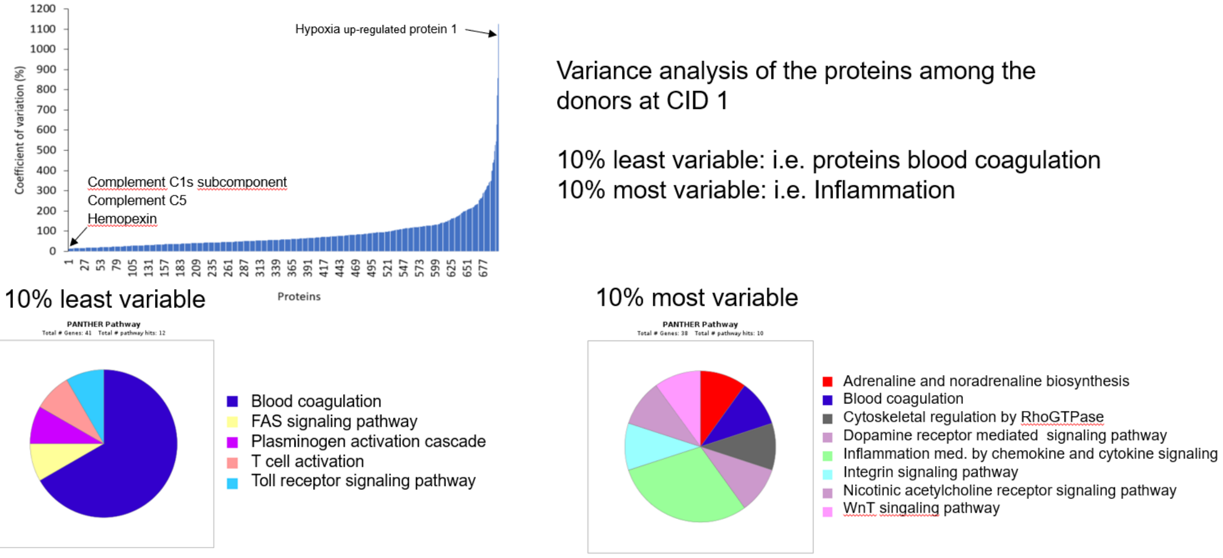

Supplemental Fig. 10: Variability of proteins at CID1.

(A) The CVs for proteins at CID1 were calculated and sorted by magnitude. (B) Gene ontology analysis of the 10% least variable proteins. (C) Gene ontology analysis of the 10% most variable proteins (DAVID Functional Annotation Bioinformatics Microarray Analysis, https://david.ncifcrf.gov).

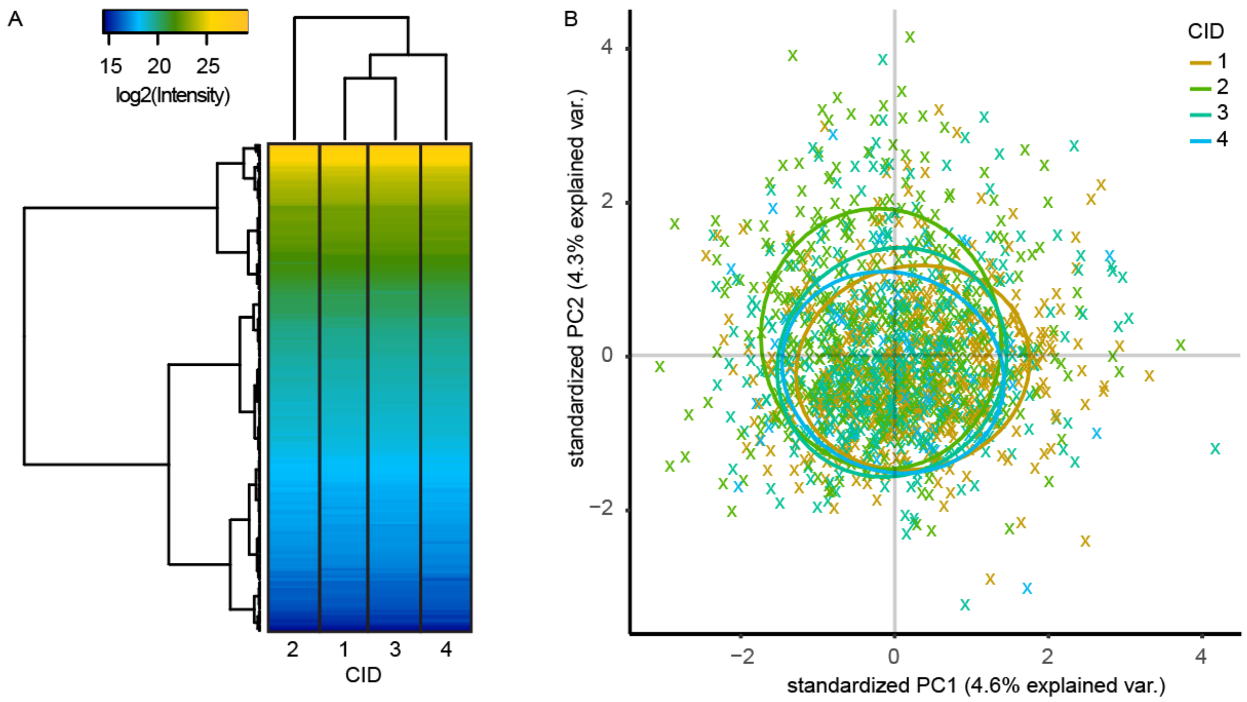

Supplemental Fig. 11: Global unsupervised clustering analysis.

(A) Proteome level unsupervised clustering and heat map visualization of the DiOGenes dataset. (B) PCA analysis of the DIA dataset with annotation of the CIDs.

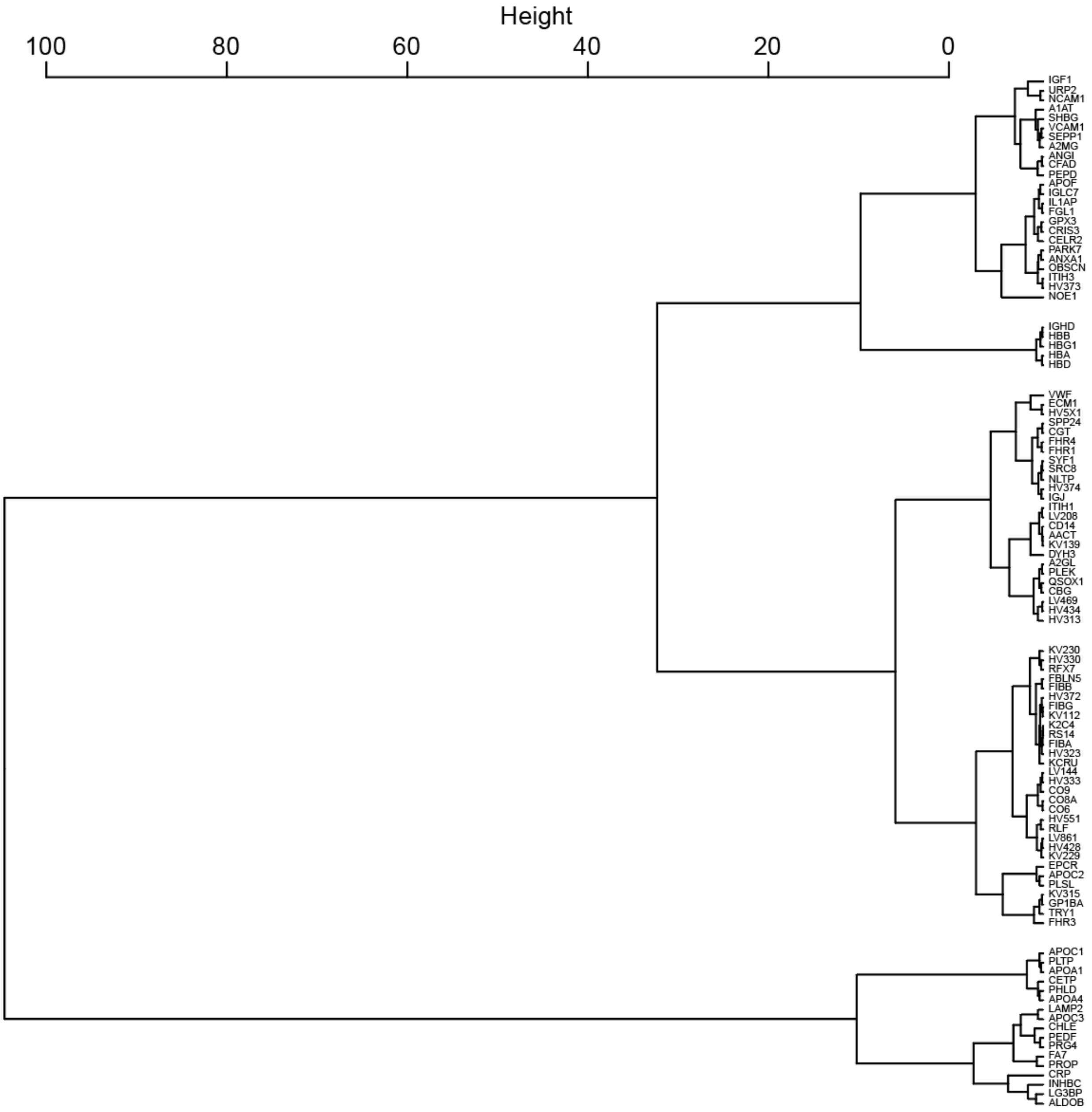

Supplemental Fig. 12: Significantly differentially abundant proteins dendrogram.

Dendrogram of the Fig. 4a with all protein names.

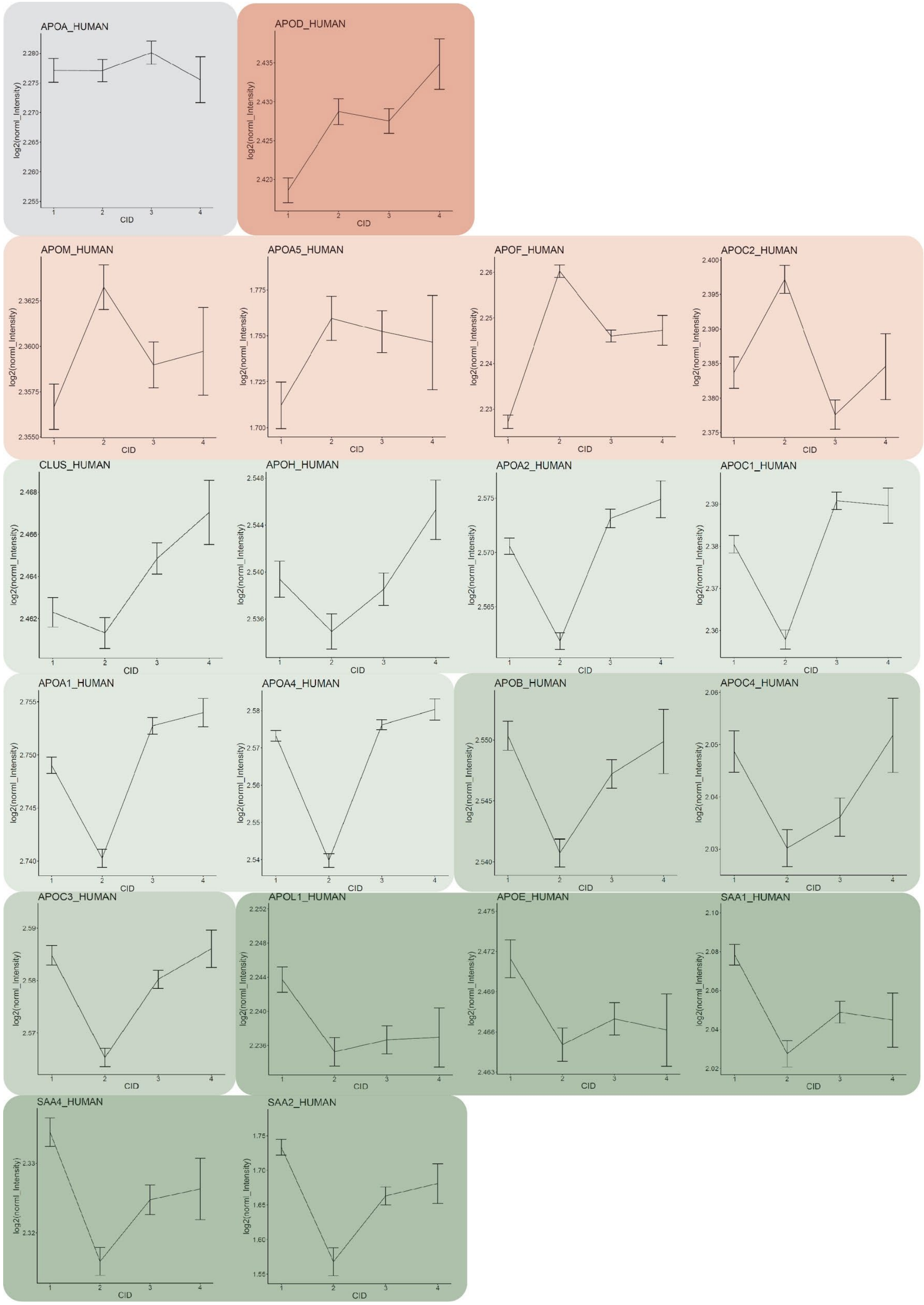

Supplemental Fig. 13: Profiles of 20 apolipoproteins profiled in the dataset. Grey marked are unchanged apolipoproteins, orange marked apolipoproteins that are increased during weight loss and green apolipoproteins reduced in weight loss.

Capillary Flow DIA on DiOGenes study

CID2 to CID1

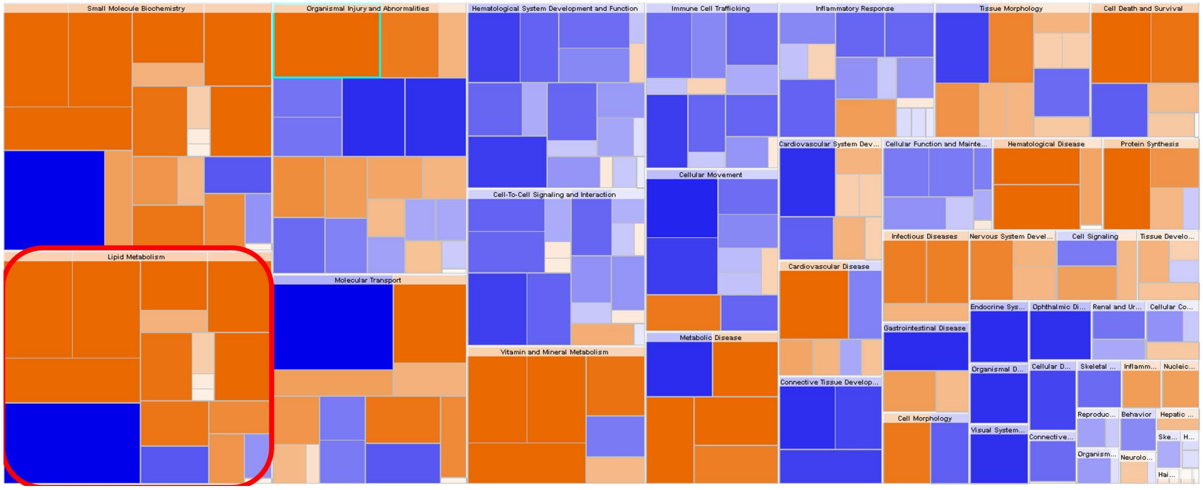

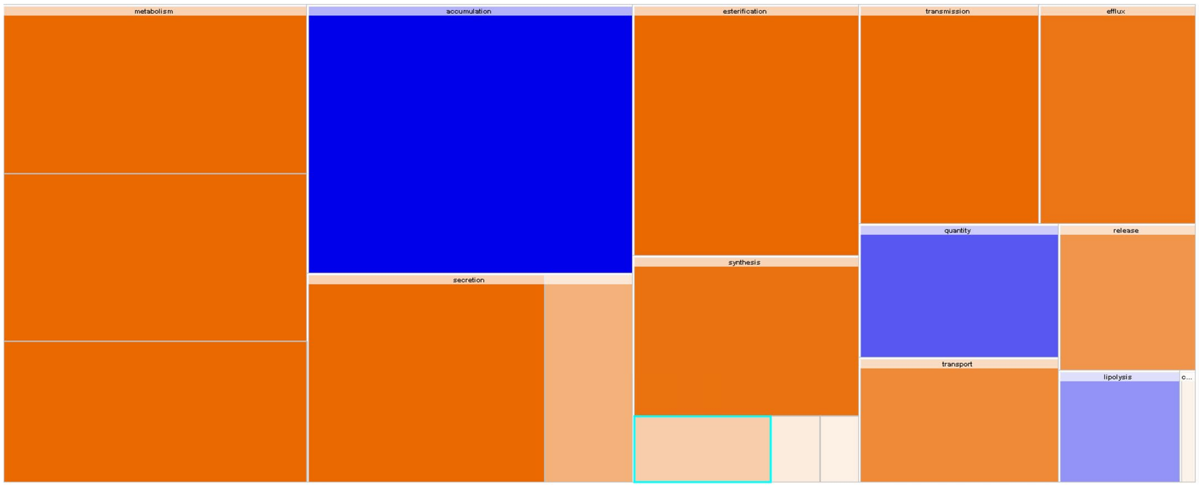

CID3 to CID1

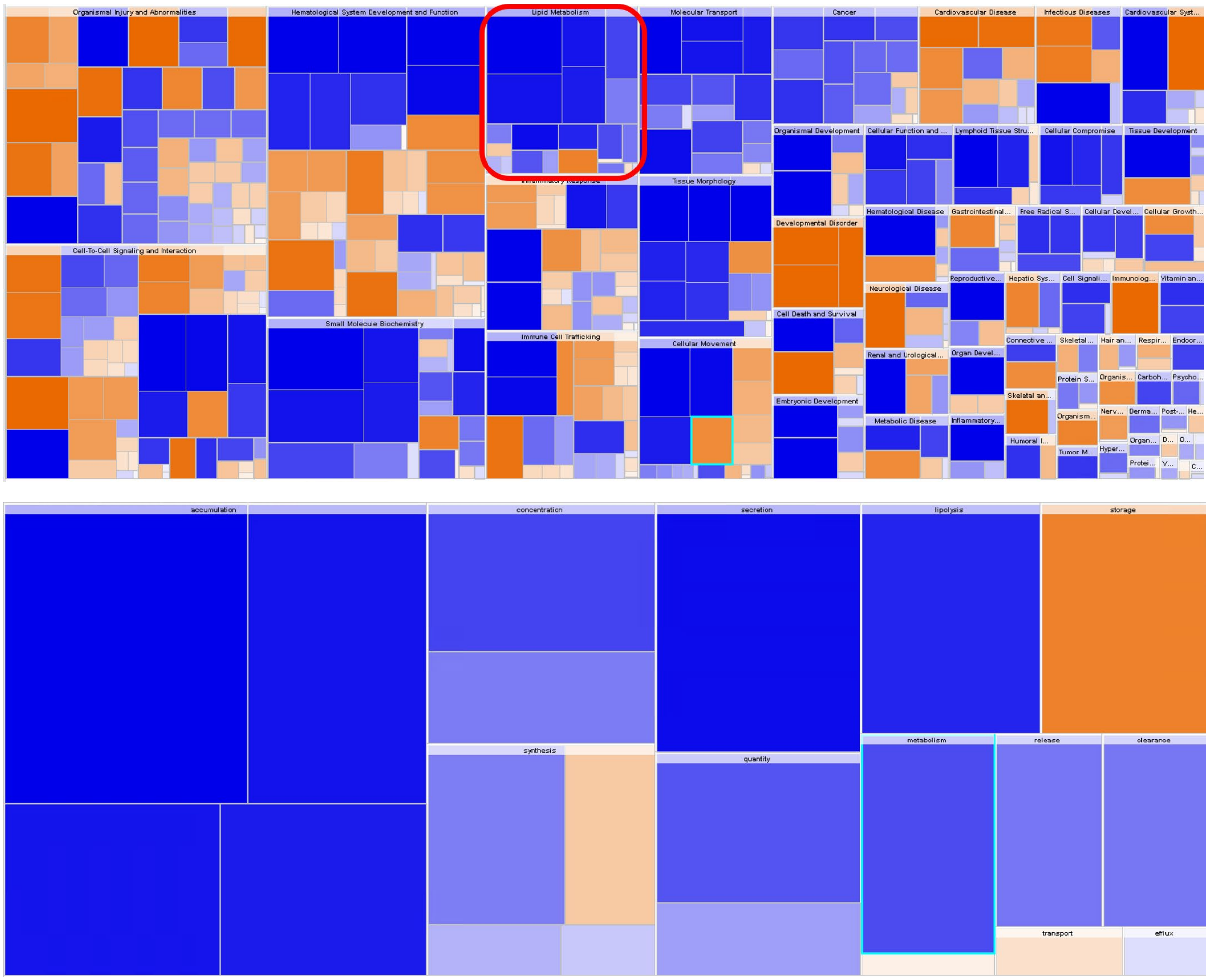

Supplemental Fig. 14: IPA tree maps.

IPA tree maps of the pairwise differential abundance tests.

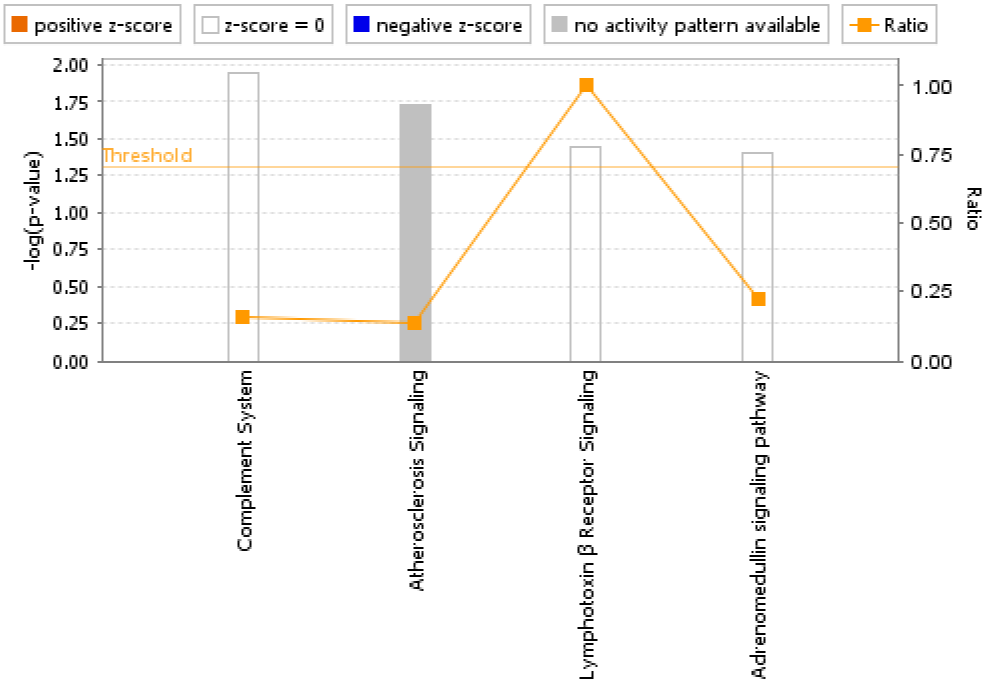

Supplemental Fig. 15: Enriched pathways IPA based on the 18 consistently changed protein in the two weight loss time points.

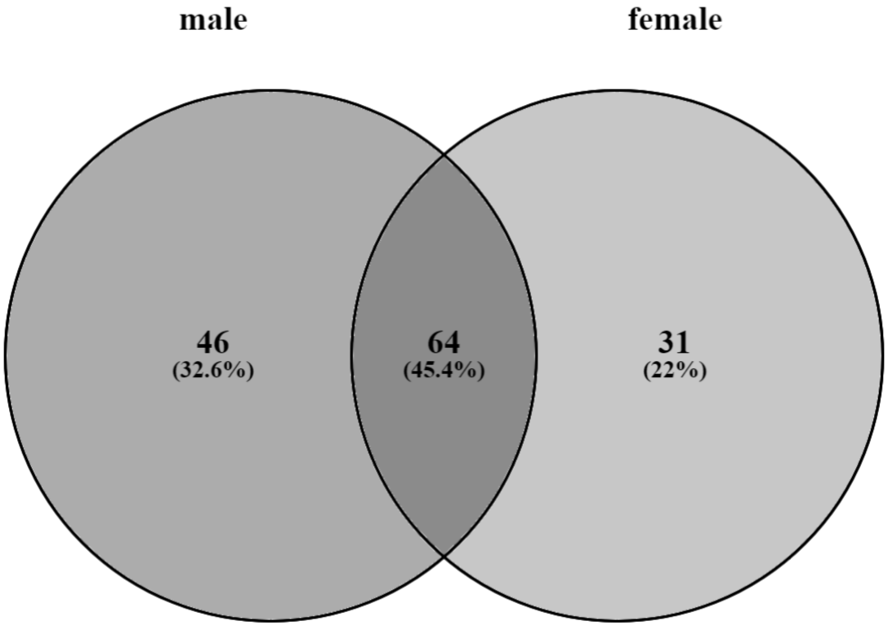

Supplemental Fig. 16: Overlap of t-test results separated by gender.
